# Spray drying siRNA-lipid nanoparticles for dry powder pulmonary delivery

**DOI:** 10.1016/j.jconrel.2022.09.021

**Published:** 2022-09-22

**Authors:** Christoph M Zimmermann, Domizia Baldassi, Karen Chan, Nathan B. P. Adams, Alina Neumann, Diana Leidy Porras-Gonzalez, Xin Wei, Nikolaus Kneidinger, Mircea Gabriel Stoleriu, Gerald Burgstaller, Dominik Witzigmann, Paola Luciani, Olivia M Merkel

**Affiliations:** 1 Department of Pharmacy, Pharmaceutical Technology and Biopharmacy, Ludwig-Maximilians Universität München, 81377 Munich, Germany; 2 Department of Biochemistry and Molecular Biology, University of British Columbia, 2350 Health Sciences Mall, Vancouver, BC V6T 1Z3, Canada; 3 NanoMedicines Innovation Network (NMIN), 2350 Health Sciences Mall, Vancouver, BC V6T 1Z3, Canada; 4 Nanotemper Technologies GmbH, Flößergasse 4, 81369 Munich, Germany; 5 Institute of Lung Health and Immunity (LHI) and Comprehensive Pneumology Center (CPC) with the CPC-M bioArchive, Helmholtz Munich, Member of the German Center for Lung Research (DZL), Munich, Germany; 6 Department of Medicine V, University Hospital, LMU Munich, Member of the German Center for Lung Research (DZL), Munich, Germany; 7 Center for Thoracic Surgery Munich, Ludwig-Maximilians-University of Munich (LMU) and Asklepios Pulmonary Hospital; Marchioninistraße 15, 81377 Munich and Robert-Koch-Allee 2, 82131 Gauting, Germany; 8 NanoVation Therapeutics Inc., 2405 Wesbrook Mall 4th Floor, Vancouver, V6T 1Z3 Canada; 9 Department of Chemistry, Biochemistry and Pharmacy, University Bern, Freiestrasse 3, Bern, Switzerland

**Keywords:** lipid nanoparticles, LNP, Onpattro®, RNA therapeutics, siRNA delivery, spray drying, pulmonary delivery, respiratory diseases, human precision-cut lung slices, formulation screening

## Abstract

While all the siRNA drugs on the market target the liver, the lungs offer a variety of currently undruggable targets which could potentially be treated with RNA therapeutics. Hence, local, pulmonary delivery of RNA nanoparticles could finally enable delivery beyond the liver. The administration of RNA drugs via dry powder inhalers offers many advantages related to physical, chemical and microbial stability of RNA and nanosuspensions. The present study was therefore designed to test the feasibility of engineering spray dried lipid nanoparticle (LNP) powders. Spray drying was performed using 5% lactose solution (m/V), and the targets were set to obtain nanoparticle sizes after redispersion of spray-dried powders around 150 nm, a residual moisture level below 5%, and RNA loss below 15% at maintained RNA bioactivity. The LNPs consisted of an ionizable cationic lipid which is a sulfur-containing analog of DLin-MC3-DMA, a helper lipid, cholesterol, and PEG-DMG encapsulating siRNA. Prior to the spray drying, the latter process was simulated with a novel dual emission fluorescence spectroscopy method to preselect the highest possible drying temperature and excipient solution maintaining LNP integrity and stability. Through characterization of physicochemical and aerodynamic properties of the spray dried powders, administration criteria for delivery to the lower respiratory tract were fulfilled. Spray dried LNPs penetrated the lung mucus layer and maintained bioactivity for >90% protein downregulation with a confirmed safety profile in a lung adenocarcinoma cell line. Additionally, the spray dried LNPs successfully achieved up to 50% gene silencing of the house keeping gene GAPDH in *ex vivo* human precision-cut lung slices at without increasing cytokine levels. This study verifies the successful spray drying procedure of LNP-siRNA systems maintaining their integrity and mediating strong gene silencing efficiency on mRNA and protein levels both *in vitro* and *ex vivo*. The successful spray drying procedure of LNP-siRNA formulations in 5% lactose solution creates a novel siRNA-based therapy option to target respiratory diseases such as lung cancer, asthma, COPD, cystic fibrosis and viral infections.

## Graphical abstract

**Graphical Abstract:**
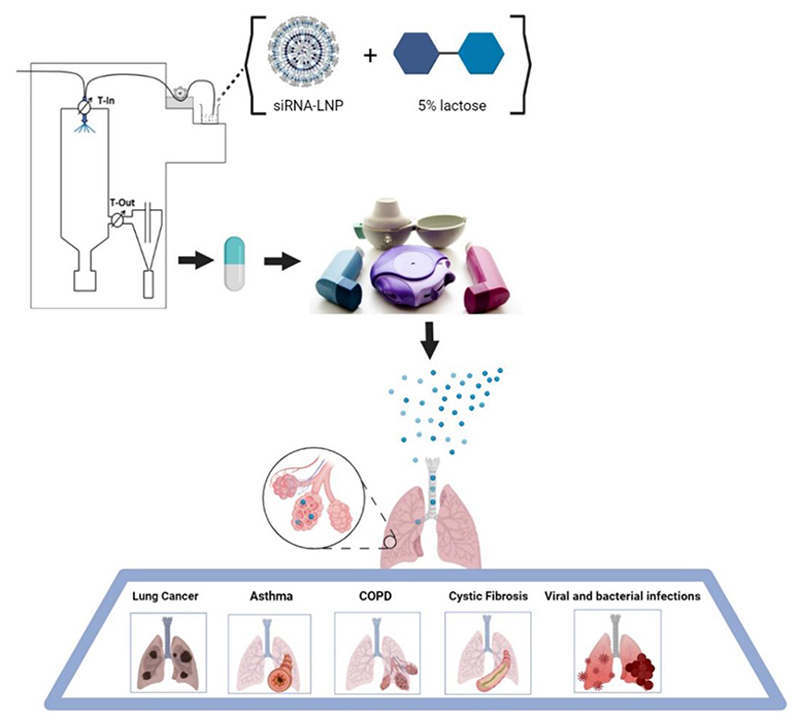

## 1 Introduction

The lungs are the most vulnerable internal organ to infection and injury from the external environment because of its constant exposure to particles, chemicals and infectious organisms in ambient air. Respiratory diseases impose an immense worldwide health burden. Altogether, more than 1 billion people suffer from either acute or chronic respiratory conditions. [1] Chronic respiratory diseases such as asthma, chronic obstructive pulmonary disorder (COPD), cystic fibrosis and lung cancer made up more than 545 million cases in 2017. Noteworthy, this number increased by almost 40% from 1990 to 2017. [2] Since the start of the Covid-19 pandemic in 2019, an increased number of pulmonary dysfunctions on top of all other pulmonary diseases was noted [3], followed by an inevitable increasing demand for novel pulmonary therapies being locally applied to the site of action. RNA therapeutics are promising for the treatment of respiratory diseases, and nucleic acid-based therapy have been studied for COPD or asthma therapies. [4–7] In comparison to conventional therapeutic approaches using proteins, peptides, small molecules or monoclonal antibodies, RNA therapeutics provide high selectivity, potency and the possibility of personalized therapy. [8, 9] Small interfering RNA (siRNA) as one class of RNA therapeutics inhibits gene expression to improve or cure disease symptoms, underlying pathologic mechanisms, and viral infections. [10] However, siRNAs are negatively charged macromolecules that do not bind to the cell surface and do not permeate through the cell membrane. Furthermore, challenges such as nuclease degradation, off-target gene silencing and immune-stimulating effects need to be addressed and resolved. [11] To overcome these limitations, siRNAs are encapsulated in a vast variety of materials including lipids, polymers, inorganic materials, proteins and combinations of the above. Lipid-based carrier systems mimicking the composition of pulmonary surfactant or the cell membrane enhance the ability to overcome the lungs’ biological barriers and reduce toxicity and antigenicity. [12, 13]

One of the biggest breakthroughs of siRNA therapeutics was the approval of the first siRNA-LNP drug, Onpattro® (Patisiran) by the FDA (Food and Drug Association, USA) and EMA (European Medicines Agency). Onpattro® consists of lipid nanoparticles (LNPs) encapsulating siRNA in its lipid matrix to treat hereditary amyloidogenic transthyretin (TTR) amyloidosis. [14, 15] The target is the liver after intravenous administration. Recently, LNP technology has also enabled the rapid development and approval of mRNA-based vaccines against COVID-19. [16] These types of LNPs consist of phospholipids, cholesterol, polyethylene glycol-conjugated lipids, and ionizable helper lipids. Ionizable helper lipids bind to anionic RNA to enable efficient encapsulation and promote endosomal escape following internalization into the target cell. [17] The major advantage of LNP technology is its adaptability to different siRNA payloads as its physicochemical properties remain similar. [18] Therefore, LNP formulations can potentially be pursued to deliver therapeutic cargoes via different administrative routes for the treatment of a large variety of diseases.

The lungs are one of the most complex organs and offers advantages of local, over systemic, delivery such as noninvasive access and a large alveolar surface area. [19] Furthermore, the administered dose can be reduced for local effects compared to systemic delivery, resulting in decreased side effects. Regarding RNA delivery to the lungs, the absence of serum proteins on the air-side keeps the nuclease activity relatively low. [20] Therefore, drugs can most effectively be delivered by inhalation and are immediately available to the lungs. [21, 22] In order to deliver drugs or nanoparticles to the lungs, incorporation into microparticles with aerodynamic diameters between 1 and 5 μm are required. The matrices of these particles need to consist of excipients, such as FDA-approved lactose [23] or mannitol [24], which readily dissolve upon contact with the lung fluid to release their cargo. [25] During the spray drying process, two main stress factors applied to the product are heat and shear forces which may tear the nanoparticle structure and result in degradation of the cargo. To avoid a negative impact on the quality of nanoparticles, appropriate sugar excipients need to be selected and the process parameters need to be optimized. Another important aspect that needs to be considered is the restricted outlet temperature range for thermolabile drugs. [26] siRNAs are prone to degrade at about 90 °C, and Onpattro-like LNPs have shown phase transition temperatures of 38 °C with a gradual phase transition.[27] Furthermore, during the development of novel dosage forms for lung administration, lung deposition, retention, dissolution, metabolism and toxicity of spray dried microparticles need to be tested. [28]

The aim of this study was to transfer LNP formulations based on an updated Onpattro® composition but consisting of a neutral, positively or negatively charged helper lipid (nLNP, (+)LNP and (-)LNP) into a successful spray dried powder at maintained physicochemical properties and siRNA integrity for pulmonary application. The target range was set to obtain sizes after redispersion of around 150 nm, a residual moisture level below 5%, and RNA loss below 15% at maintained RNA bioactivity. A novel dual emission fluorescence spectroscopy method was used to simulate the stability of LNPs in different excipient solutions (PBS, lactose, mannitol and trehalose) at different temperatures. This prescreening to find the best combination of LNPs, excipients and temperatures for the spray drying process, supersedes the necessity for a trial-and-error approach. Quantification of siRNA and lipid concentration of LNPs after redispersion of spray dried powders underlines a quality criterion which should always be carried out to avoid performance loss or increased material costs. The particles’ performance and siRNA integrity were tested *in vitro* in lung cancer cells expressing enhanced green fluorescent protein (eGFP), and *ex vivo* in human precision-cut lung slices (hPCLS) targeting the house keeping gene GAPDH. The findings of this study provide unique insights into the possibility of spray drying siRNA embedded LNPs and maintaining their bioactivity while keeping optimal properties for pulmonary delivery.

## 2 Materials & Methods

### 2.1 Materials

Dicer substrate double-stranded siRNA targeting green fluorescent protein (DsiRNA EGFP, 25/27) (siGFP), dicer substrate double-stranded siRNA targeting the house-keeping gene GAPDH (DsiRNA GAPDH) (siGAPDH) and scrambled, non-specific control (siNC) were purchased from IDT (Integrated DNA Technologies, Inc., Leuven, Belgium) (Table S1). [29–31] Cholesterol, tris-EDTA buffer solution 100x (T9285), RPMI-1640 medium (R8758), fetal bovine serum (FBS) (F9665), penicillin-streptomycin (P/S) (P4333), G418 disulfate salt solution (G8168), Dulbecco’s phosphate buffered saline (D-PBS) (D8537), isopropanol for molecular biology, methylthiazolyldiphenyl-tetrazoliumbromid (MTT), D-mannitol, HEPES buffer 1M, Triton X-100, MISSION® siRNA flourescent universal negative control #1, cyanine 4 (SIC005) and cholesterol quantitation kit were purchased from Sigma-Aldrich, a subsidiary of Merck KGaA (Darmstadt, Germany). PEG-DMG, DSPC, DSPG and DOTAP were bought from Avanti Polar Lipids, Alabaster, USA. The ionizable cationic lipid is a sulfur-containing analog of DLin-MC3-DMA (pKa 6.3-6.6) defined by the structure shown in Figure S1. [32] InhaLac®230, lactose monohydrate for dry powder inhalers, was purchased from Meggle Group (Wasserburg, Germany). Quant-it™ RiboGreen DNA reagent, chloroform as molecular biology reagent, black and white 96-well plates (10307451), TRIzol reagent, Power SYBR™ green PCR master mix and Aquastar® water standard oven 1% were bought from Thermo Fisher Scientific (Schwerte, Germany). Pumpsil® tubings were bought from Watson-Marlow GmbH (Rommerskirchen, Germany) and had an inner diameter and a thickness of 1.6 mm. Lysing matrix D 2.0 mL tubes and D(+)-trehalose were purchased from VWR International GmbH (Ismaning, Germany). White 96 well PCR plate and 0.2 mL PCR tubes were purchased from Biozym Scientific GmbH (Hessisch Oldendorf, Germany). AL-crucibles at 40 μL (ME-26763) for DSC measurements were bought from Mettler Toledo (Fürstenfeldbruck, Germany). High sensitivity capillaries (PR-C006) produced by NanoTemper Technologies GmbH were used for all formulation screening measurements.

### 2.2 Preparation of lipid nanoparticles (LNPs) entrapping siRNA

LNP-siRNA formulations had a lipid composition based on the clinically approved Onpattro® formulation and were prepared as previously described [14, 15, 33]. Briefly, lipid components (ionizable cationic lipid, helper lipid, cholesterol, and PEG-DMG) at molar ratios of 50:10:38.5:1.5 mol% were dissolved in ethanol to a concentration of 10 mM total lipid. Different helper lipids, i.e. 1,2-distearoyl-*sn*-glycero-3-phosphocholine (DSPC), 1,2-dioleoyl-3-trimethylammonium-propane (DOTAP), and 1,2-distearoyl-sn-glycero-3-phospho-(1'-rac-glycerol) (DSPG), were used to enable formation of LNP-siRNA systems with near neutral [nLNP], positive [(+)LNP], and negative [(-)LNP] zeta potential, respectively. Purified siRNA (siNC, siGFP and siGAPDH) was dissolved in 25 mM sodium acetate pH 4 buffer to achieve an N/P ratio of 3, which is the charge ratio between the ionizable cationic head group on the lipid to the anionic phosphate in the RNA backbone. The two solutions were mixed through a T-junction mixer at a total flow rate of 20 mL/min, and a flow rate ratio of 3:1 v/v (aqueous:organic phase). The resulting LNP suspension was subsequently dialyzed overnight against PBS pH 7.4, sterile filtered (0.2 μm), and concentrated to 1.0 mg/mL siRNA.

### 2.3 siRNA loading of preformed LNPs for thermal stability measurements

10 nmol MISSION® siRNA Fluorescent Universal Negative Control #1, Cyanine 5 (Cy5-siRNA) was diluted to 1000 μg / μL in 10 mM HEPES buffer pH 7.4 and used as a stock in all experiments. To load preformed LNPs, Cy5-siRNA was diluted 1:10 in 25 mM sodium acetate buffer pH 4. Afterwards, 1.2 μL LNP, 0.7 μL 100 μg / mL Cy5-siRNA and 1.4 μL 25 mM sodium acetate buffer pH 4 were gently mixed and incubated at room temperature in the dark for 5 min. LNPs were then diluted in 97 μL final formulation buffer (PBS, 5 % mannitol, 5 % trehalose or 5 % lactose) and incubated for additional 30 min at room temperature in the dark before being further analyzed.

### 2.4 Thermal stability of LNPs

A novel and highly sensitive method was used to characterize environmental changes experienced by Cy5- labelled siRNA within different LNPs in regards to their excipient buffer and temperature. The system used was a prototype instrument developed by NanoTemper Technologies GmbH to monitor a dedicated dual-emission optical system. Detection channels were used at LED excitation of 570 nm and emission detection with two filters simultaneously, one at 640 +/- 20 nm and one at 697 +/- 29 nm.

#### 2.4.1 Fluorescence spectral detection of LNP spectral shift

Experiments were performed in a JASCO FP-8300 Fluorescence Spectrometer with a PCT-818 Peltier Temperature Controller controlled by the Spectra Manager 2.0 (JASCO Deutschland GmbH, Pfungstadt, Germany). For each sample, 100 μL LNP suspension was loaded into a quartz microcuvette and fluorescence spectra was recorded at an excitation wavelength of 590 ± 20 nm and the fluorescence emission was recorded at a wavelength between 620 – 725 nm, with a 10 nm bandgap, 1 nm step, 0.2 s integration time, using high sensitivity, with 4 accumulations per scan. The emission spectra were recorded as the temperature ramped from 20°C to 90 °C, at a ramp rate of 2 °C / min, with scans taken every 10 °C.

#### 2.4.2 Fluorescence based temperature stability scans

Thermal stability experiments were performed in coated high sensitivity capillaries (PR-C006) on a prototype instrument equipped with dual emission optics (NanoTemper Technologies GmbH, Munich, Germany). Each data point requires 10 μL of sample. For each ratiometric reading, the fluorescence was recorded simultaneously at 650 nm and 670 nm after excitation with an amber LED. For fluorescence stability experiments, data were recorded in a modified version of Pr.Control. The excitation power was set to 100 % and scans were performed from 15°C to 110 °C, with a 1 °C / min ramp rate. The temperature stressing experiments were performed in a modified version of Pr.TimeControl, with the temperatures as indicated in the figures, and a temperature ramp rate of 7 °C / min.

### 2.5 Spray drying of LNPs

For production of spray dried LNPs, a B-290 spray drying tower (Büchi Labortechnik, Flawil, Schweiz) was used. Pumpsil Tubing 1.6 mm x 1.6 mm (Watson Marlow Tubing, Falmouth, UK) with a pump rate of 1.4 mL/min was chosen. Nitrogen functioned as atomizing gas, whereas drying gas was air. In order to avoid dust and other airborne particles, both nitrogen gas and air were filtered through a 0.2 μm PTFE membrane pore. Pressurized air was used to ensure sufficient heating of the air supply and to avoid overheating of the Büchi’s vacuum pump. The aspirator was set to 70% and vacuum ranged from -40 mbar to -35 mbar. The airflow was set to 40 mm corresponding to 473 NL/h. The inlet-temperatures (T-In) were set to 100 °C and 120 °C resulting in measured outlet-temperatures (T-Out) of accordingly 62 °C and 72 °C ± 2 °C, respectively. Each individual stock solution of LNP-siRNA formulations was diluted to a concentration of 30 μg siRNA in 5000 μL of a specified solvent (highly purified water (HPW) with lactose (InhaLac) at 5% w/V, sterile filtered). This resulted in an siRNA to sugar concentration of 0.12 μg siRNA/mg lactose.

### 2.6 Loss detection after spray drying of LNPs

#### 2.6.1 siRNA quantification

The Quant-IT™ Ribogreen assay was adapted as described in Walsh et al. [34] Briefly, LNPs were either freshly prepared or redispersed as described above. For each reading, 50 μL of samples was transferred in a black 96-well plate and filled to 100 μL with 2% Triton X-100 solution. A siRNA standard curve was pipetted at 25.0, 10.0, 5.0 and 2.5 μL of a stock solution (20 μg/mL) resulting in final concentration of 2.5, 1, 0.5 and 0.25 μg/mL, respectively. The plate was incubated at 37°C for 30 min in a shaking incubator. Upon the addition of the Ribogreen reagent at a 1:100 dilution, the fluorescence intensities were measured at an excitation wavelength of 480 nm and an emission wavelength of 525 nm. The siRNA loss was quantified by normalizing the siRNA amount of spray dried samples to the siRNA amount of fresh LNP samples.

#### 2.6.2 Cholesterol quantification

The cholesterol quantification method was performed following the Cholesterol Quantitation Kit product information sheet (Sigma-Aldrich). To construct the standard curve, 20 μL of a 2 μg/μL cholesterol standard solution was diluted with 140 μL of the cholesterol assay buffer resulting in a 0.25 μg/μL stock solution. Amounts of 0, 4, 8, 12, 16 and 20 μL were pipetted into a clear 96 well plate, and each well was replenished to 50 μL by addition of cholesterol assay buffer. To measure the cholesterol content of the test samples, 50 μL per sample were transferred into the well plate. Since the cholesterol amount was unknown, each individual sample was diluted several times and replenished to 50 μL with cholesterol assay buffer. Subsequently, the Reaction Mix at 50 μL/well was prepared and added to each well. The plate was protected from light and placed in a shaking incubator for 60 min at 37°C. The cholesterol concentration was determined by measuring the absorbance at 570 nm.

### 2.7 Hydrodynamic diameter and zeta (ζ) potential measurements of LNPs

Hydrodynamic diameters and polydispersity indices (PDI) were measured in disposable cuvettes (Brand GmbH, Wertheim, Germany) using the Zetasizer Nano ZS instrument (Malvern Instruments Inc., Malvern, U.K.). To measure the size and PDI of spray dried formulations after redispersal, approximately 8.33 mg of spray dried LNP powder was dissolved in 100 μL HPW. This equates to 1 μg of siRNA (10 μg siRNA/mL). For comparison, fresh LNPs (c = 1 mg/mL) were diluted in 5% lactose to reach a concentration of 10 μg siRNA/mL. All samples were detected at a backscatter angle of 173°. Results are presented as average size (nm) ± SD. Zeta potentials were measured by Laser Doppler Anemometry (LDA) using a Zeta Cell (Zetasizer Nano series, Malvern, UK) containing a 6.5X dilution of the same 100 μL sample of LNP suspension. For each LNP formulation, measurements were presented as an average charge (mV) ± SD.

### 2.8 Residual water content – Karl Fischer titration

The residual water content for spray dried LNPs in 5% lactose (w/V) was determined by weighing 10 mg powder of each LNP sample into 2R vials. A 1% water standard was equally prepared with approximately 40-50 mg powder. Empty vials served as blank values. For coulometric measurements, an Aqua 40.00 Karl Fischer Autosampler-Titrator with corresponding software from Analytik Jena AG (Jena, Germany) was used. The oven was heated to 100°C, and the final drift was set to less than 10.0 μg/min. Blank measurements were run and automatically subtracted from the standards and samples. Residual moisture measurements were considered valid if the 1% water standard measurement resulted in a value between 0.9 and 1.1%. Results are presented as mean residual moisture (%) ± SD.

### 2.9 Differential Scanning Calorimetry (DSC)

For calorimetric measurements 5 to 10 mg of spray dried LNPs were weighed into AL-crucibles at 40 μL volume and closed. The reference was an empty crucible. The reference and samples were inserted into the oven at a set point of 25 °C. Measurements were taken with a DSC 214 Polyma (Erich NETZSCH GmbH & Co. Holding KG, Selb, Germany) starting from 0°C with a ramp of 8 °C/min until temperature reached 200 °C for all spray dried LNP formulations in 5% lactose (w/V) and spray dried 5% lactose (w/V). Data was analyzed using the Proteus Analysis software.

### 2.10 Scanning Electron Microscopy (SEM)

Scanning electron microscopy (SEM) is used to determine the geometric diameter and morphology of spray dried powders. A small amount of spray dried LNPs was placed on top of a stub covered with double-sided carbon tape. The stub was then coated with carbon under vacuum for 40 s. The microparticles were examined imaged using a FEI Helios G3 UC (Thermo Fisher Scientific, Schwerte, Germany).

### 2.11 Aerodynamic properties of spray dried LNPs

For the analysis of the aerodynamic properties of spray dried powders, procedures specified in the monograph 2.9.18, apparatus E, of the European Pharmacopoeia was performed using a next generation impactor (NGI) from Copley Scientific (Nottingham, UK). The measurement procedure was adapted as previously described. [7] Spray dried LNP powder was transferred into 2-3 hydroxypropylmethylcellulose capsules. Each capsule was loaded into a Handihaler® (Boehringer Ingelheim Pharma GmbH & Co. KG, Ingelheim, Germany) and hole-punched. Every capsule was discharged twice with a 5 s interval between the two actuations. Following the application of the Handihaler®, the same procedure as in 2.6.1. was performed. Every stage of the NGI was washed with 2% Triton-X buffer. The induction port (IP) was washed with 5 mL and the pre-separator (PS) was pre-filled with 15 mL 2% Triton-X buffer. The small cups were filled with 2 mL 2% Triton-X buffer, whereas the greater cups were filled with 4 mL 2% Triton-X buffer solution. All parts were cautiously shaken and placed on a horizontal shaker for 20 min. A standard curve of fresh siRNA was prepared and topped up to 100 μL with 2% Triton-X buffer. As a control, fresh LNPs, at siRNA concentration of 10 μg/mL, similar to the redispersed samples, were prepared in 2% Triton-X buffer. Three aliquots of 100 μL from each stage were used for further analysis. All samples were pipetted to a black 96-well plate and put into a shaking incubator for 60 min at 37 °C. Upon the addition of Ribogreen reagent at a 1:100 dilution, the fluorescence intensities were measured at an excitation wavelength of 480 nm and an emission wavelength of 525 nm. The mass median aerodynamic diameter (MMAD), geometric standard deviation (GSD), fine particle dose (FPD), fine particle fraction (FPF) and powder recovery (%) were calculated as described in the European Pharmacopoeia considering fine particles at sizes below 5 μm MMAD.

### 2.12 Mucus penetration assay of spray dried LNPs

The mucus penetration of fresh versus spray dried LNPs was adapted from Casciaro et al. [35] Briefly, 75 μL of artificial mucus was transferred onto 8 μm pore polycarbonate membrane Transwell® inserts submerged in 300 μL of acceptor medium in a 24-well plate. Afterwards, 5 mg of spray dried LNPs was redispersed in HPW (0.6 μg siRNA / 100 μL) and labelled with 1 μL 1,1-dioctadecyl-3,3,3,3-tetramethylindodicarbocyanine solution (DiD) solution, acting as a fluorescence lipid marker. The same procedure was performed to assess diffusion of fresh LNPs at the same concentration. Hence, 100 μL of samples was deposited on artificial mucus. Non-deposited samples were stored for 24 h under light exclusion for further fluorimetric analysis as a comparable value. Simulated interstitial lung fluid (SILF) was used as acceptor media for mucus diffusion experiments and placed on the bottom of the well, respectively. SILF was carefully prepared according to the instructions provided by Moss et al. [36] At scheduled time intervals (0.5, 1, 2, 4 and 24 h), the acceptor medium was collected, pipetted to a 96 well plate and quantified by spectrofluorimetric analysis at excitation wavelength of 520 nm and an emission wavelength of 635 nm. Values were calculated by normalizing each mucus deposited sample value to the non-deposited and stored DiD-LNPs. The results are reported as percentage (%) of total LNPs permeated over time.

### 2.13 *In vitro* characterization of spray dried LNPs in a lung cell line

#### 2.13.1 Cell Culture

The human non-small cell lung carcinoma cell line H1299 (ATCC CRL-5803) stably expressing enhanced green fluorescence protein (eGFP) was cultured in RPMI 1640 medium supplemented with 10% FBS, 1% P/S and 0.4% G418. Cells were passaged every 3 days with 0.05% v/v trypsin and subcultured in 75 cm^2^ flasks. H1299-GFP cells were kept in a humidified atmosphere at 37 °C with 5% CO_2_.

#### 2.13.2 *In vitro* GFP protein downregulation

To evaluate the *in vitro* gene silencing efficiency, H1299-GFP cells were seeded in a 24-well plate at a density of 2.5x10^4^ cells per well in 500 μL medium at 37 °C and 5% CO_2_. Fresh LNPs of different helper lipids were prepared at concentrations of 1 μg/mL and 10 μg/mL in 5% lactose (w/V). Comparably, 0.833 mg and 8.333 mg of spray dried LNPs encapsulating siNC or siGFP, were resuspended in 100 μL HPW resulting in equal concentrations as aforementioned. The day after, 100 μL of each sample was added to 400 μL of fresh culture medium and incubated for 24 h at 37 °C and 5% CO_2_. The medium was then discarded and replaced with 500 μL of fresh medium, and the plates were further incubated for another 24h. At the end of the incubation time, cells were washed with PBS, trypsinized and collected. After centrifugation at 400 rcf for 5 min, the supernatant was discarded and the cell pellet was washed two times in PBS before being resuspended in PBS with 2 mM EDTA. Samples were analyzed by flow cytometry (Attune® NxT, Thermo Fischer Scientific, Waltham, Massachusetts, USA), and the median fluorescence intensity (MFI) of GFP protein expression was measured by using a 488 nm excitation laser and the emitted light passing through a 530/30 nm band pass emission filter set (BL-1H) was detected. All LNPs samples were gated by morphology for a minimum of 10,000 viable cells. Results are displayed as mean MFI values (%) ± SD.

#### 2.13.3 *In vitro* cytotoxicity of spray dried LNPs

Cell viability after transfection with spray dried LNPs (neutral, positive and negative LNPs) was tested via an MTT Assay as described previously.[37, 38] Shortly, 5,000 H1299-GFP cells per well were seeded in 100 μL medium onto a transparent 96-well plate (BioLite 96 well multidish, Thermo Fisher Scientific, Rochester, New York, USA). The samples were prepared by redispersing 0.833 mg (1 μg siRNA) and 8.33 mg (10 μg siRNA) of spray dried powders in 100 μL of HPW. After 24 h, 90 μL of prewarmed medium was added to each well and supplemented with 10 μL of sample, respectively. Thus, siRNA concentrations of 1.0 and 10.0 μg/mL were added for each LNP sample. The plate was incubated for 24 h at 37 °C and 5% CO_2_. As a full viability control, cells were incubated in 100 μL of liquid consisting of 10 μL sterile 5% lactose solution (m/V) and 90 μL medium. After 24 h, the media was aspirated and 200 μL of MTT containing medium (0.5 mg/ml in serum-free RPMI-1640 medium) was added to each well. Cells were incubated for another 3 h at 37 °C and 5% CO_2_. Subsequently, the cell culture medium was completely removed, and insoluble purple formazan crystals, converted from water soluble MTT by metabolically active mitochondria, were dissolved in 200 μl DMSO. [39] The plate was set on a horizontal shaker for 20 min for all crystals to dissolve. The absorbance was measured at 570 nm, corrected with background values measured at 680 nm, using a microplate reader (TECAN Spark, TECAN, Maennedorf, Switzerland). The data are shown as mean ± SD as percentage of viable cells in comparison to untreated cells representing 100% viability.

### 2.14 *Ex vivo* gene silencing of spray dried LNPS in human precision-cut lung slices (hPCLS)

#### 2.14.1 Human tissue, ethics statement and hPCLS

Human tissue was obtained from the CPC-M bioArchive at the Comprehensive Pneumology Center (CPC), from the University Hospital Großhadern of the Ludwig-Maximilian University (Munich, Germany) and from the Asklepios Biobank of Lung Diseases (Gauting Germany). Participants provided written informed consent to participate in this study, in accordance with approval by the local ethics committee of the Ludwig-Maximilians-Universität Munich, Germany (Project 19-630). Precision-cut lung slices (PCLS) were prepared as described before. [40–42] Briefly, PCLS were prepared from tumor-free peri-tumor tissue. The lung tissue was inflated with 3% agarose solution and solidified at 4°C. Afterwards, 500 μm-thick slices were cut from tissue blocks using a vibration microtome (HyraxV50) (Karl Zeiss AG, Oberkochen, Germany). PCLS were cultured in DMEM F-12 medium supplemented with 0.1% FBS. Prior to the experiments, PCLS were cut by means of a biopsy puncher into 4 mm-diameter PCLS punches.

#### 2.14.2 LNP transfection, nucleic acid extraction and qPCR

For the gene silencing of hPCLS cells, LNPs loaded with siGAPDH and siGFP were spray dried in 5% lactose. The spray dried powder was redispersed to reach a concentration of 10 μg siRNA/mL. 100 μL of each sample was added to a well consisting of three PCLS punches in 500 μL medium, respectively. The plate was incubated for 24 h at 37 °C and 5% CO_2_. Once the incubation time was completed, the RNA extraction was performed by homogenizing the hPCLS punches in 1 mL TRIzol using a Fast Prep 24 Tissue Lyzer (M.P. Biomedicals, Irvine, California, USA). The samples were incubated for 5 min at room temperature. Subsequently, 0.2 mL of chloroform was added, and each tube was vigorously mixed, followed by another incubation at room temperature for 3 min. Afterwards, samples were centrifuged at 11000 rcf for 15 min at 4 °C. The aqueous phase containing RNA was transferred into a new tube. Next, 500 μL of isopropanol was added and incubated for 10 min at room temperature before another centrifugation at 11000 rcf for 10 min at 4 °C was run. The supernatant was discarded, and the pellet was washed with 1 mL of ice-cold 75% ethanol followed by centrifugation at 7500 rcf for 5 min at 4°C. The supernatant was discarded and the RNA pellet was resuspended in 30 μL of RNase free water. The RNA concentration and purity were quantified by RT-qPCR. In brief, cDNA was synthesized from total RNA using high-capacity cDNA synthesis kit (Applied Biosystems, Waltham, Massachusetts, USA). The obtained cDNA was then diluted 1:10, and a qPCR was performed using the SYBR™ Green PCR Master Mix (Thermo Fischer Scientific, Waltham, Massachusetts, USA) with primers for human GAPDH and β-actin (Qiagen, Hilden, Germany) for normalization. Cycle thresholds were acquired by auto setting within qPCRsoft software (Analytic Jena AG, Jena, Germany). Three individual batches of spray dried LNPs (siGAPDH and siGFP) were tested on three individual donor hPCLS samples. The GAPDH silencing results are reported in the mean percentages (%) normalized to siGFP values ± SEM.

#### 2.14.3 Cytokine secretion from hPCLS

To assess the toxicity of the spray dried LNPs toward human lung tissue, the levels of 12 pro-inflammatory cytokines in the supernatant of the treated hPCLS was determined using the human LEGENDplex Inflammation Panel 1 kit (BioLegend, San Diego, USA) according to the manufacturer’s protocol. Briefly, 25 μL samples of supernatant were diluted with the supplied assay buffer and incubated with the supplied beads for 2h. After washing the beads, they were incubated for 1 h with the detection antibodies, and the fluorophore was added. After further incubation, the beads were washed and diluted in PBS/EDTA buffer for analysis on an Attune® NxT flow cytometer. Cytokine levels were determined relative to a standard curve obtained with a standard supplied by the manufacturer.

### 2.15 Statistics, data analysis and presentation

All experiments were run in independent triplicates. Experimental data was analyzed for statistical significance using the One Way or Two Way ANOVA repeated measurements on the GraphPad Prism 5 software with either Bonferroni or Dunnetts post-hoc test with p>0.05 considered not significant (ns),, * p<0.05, **p<0.01, ***p<0.001. Data analysis was performed using Python (3.8.8) using the ipython (v7.29.0), matplotlib (v3.5.0), numpy (v 1.20.3), seaborn (v 0.11.2) and GraphPad Prism 5 data science packages. Analysis routines and algorithms were specifically written to analyze dual emission fluorescence traces.

## 3 Results and Discussion

### 3.1 LNP stability using dual emission fluorescence spectroscopy

Lipid based nanoparticles are most commonly spray dried at comparably low temperatures because the lipid component acts as a limiting factor showing phase transition temperatures at about 55°C for the used helper lipids. [43] In comparison, when spray drying polymer-based nanoparticles, the polymers can resist much higher temperatures having melting points over 100°C. In this case, the cargo might face degradation or melting before the polymer does. [7] Therefore, spray drying lipids requires a maximum temperature which does not melt the lipid layers while still reducing the moisture levels to a sufficient level in the produced powders, as this is important for avoiding agglomeration and microbial growth. However, the residual moisture level depends on the excipient used and the temperature applied. It has been shown that spray dried powders based on crystalline excipients such as mannitol can be obtained with very low residual moisture level below 0.5%. In comparison, amorphous sugars such as lactose and trehalose ensue higher residual moisture levels of 3 – 5% after spray drying. [44–46] Here, we used a new dual-emission epifluorescence setup to screen all LNP formulations at different temperatures and in different excipient solutions to avoid a trial-and-error approach of selecting spray drying temperatures and finding suitable excipient solutions (Figure S2). The fluorescence-based stability measurements were found to be highly sensitive when characterizing environmental changes experienced by fluorophore-labelled siRNA within LNP formulations.

**Figure 1:**
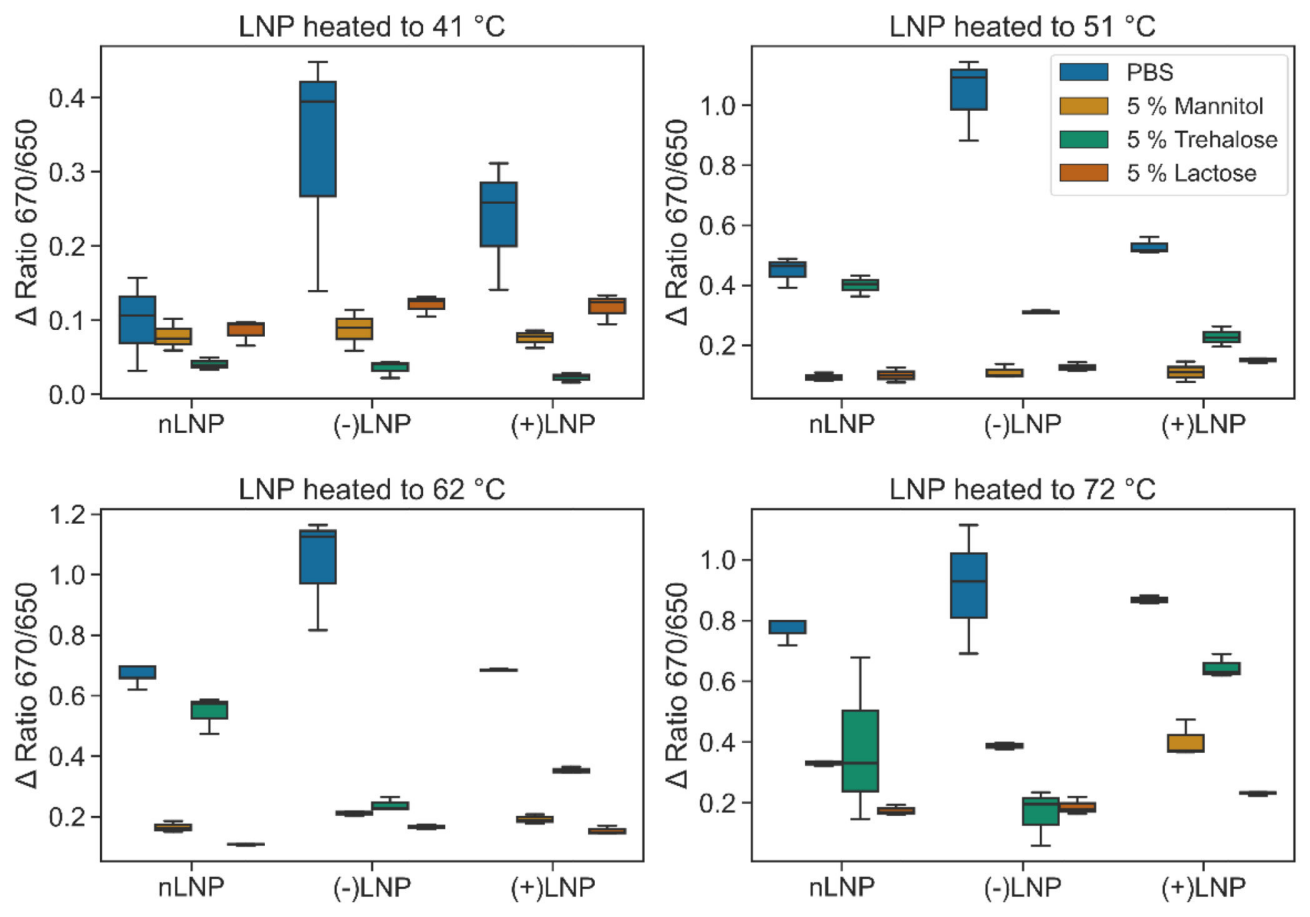
Box plots of the Δ R values observed by each LNP in different buffers (as indicated by colored legends) and hold temperatures).

Since the Cyanine 5 (Cy5) dye is very sensitive to environmental changes, its fluorescence emission is red shifted with a 6 nm peak shift from 660 to 666 nm upon encapsulation within a lipid nanoparticle (LNP) (Figure S3-S5). Instead of measuring the full emission spectrum, the fluorescence is recorded simultaneously only at two pre-selected wavelengths, 670 nm and 650 nm, with photon-multiplier-tubes (PMTs), which greatly enhance sensitivity. The small hypsochromic (blue-) or bathochromic (red-) shifts of the emission peak of the fluorescent dye are translated into large changes in the 670 / 650 nm ratio (Figure S6). These changes are then monitored as a function of temperature, either online, or after temperature stressing. Therefore, we expected to obtain information through fluorescence emission about the LNPs’ stability related to temperature applied and excipient used. All LNP samples were produced encapsulating Cy5-siRNA and monitored for a change in fluorescence ratio while heating the samples from 15°C to 110°C. LNPs were prepared in either a control PBS buffer, or in 5 % mannitol, 5 % trehalose and 5 % lactose solution (m/V) (Figure S7). The behavior of the LNPs when subjected to a melting curve is more difficult to interpret than a standard nanoDSF analysis of protein melting. Proteins have sharp melting curves where the maximum of the first derivative of the melting curve can provide a Tm of melting. LNPs, where the lipids and cholesterols have wider glass transitions as opposed to defined transition temperatures, as points of denaturing, lead to less defined curves.[27] While melting events are somewhat visible in PBS, in the sugar solutions the melting events occur over a much larger temperature range. The first derivatives of each curve were analyzed and plotted in Figure S8. The inflection temperatures (*T*_i_) do not accurately represent the stability of the LNPs, as visual inspection of the data shows that the nLNPs in 5% trehalose are likely to be more stable than an inflection of approx. 20 °C indicates. Visually, it appears that LNPs in 5 % lactose and 5 % mannitol seem to be more stable than in PBS or 5 % trehalose. An alternative approach to observe LNP behavior was sought, which would also align with spray drying methods. Here, LNPs were subjected to four temperatures (41 °C, 51 °C, 62 °C, 72 °C) mimicking the elevated outlet temperatures in a spray dryer, and then returned to the original temperature (Figure S9A). As the LNPs are heated, the ratio, and therefore the environment experienced by the Cy5-siRNA irreversibly changes. This change is quantified by the difference between the initial ratio (*R_i_*) and final ratio (*R*_I_) measurements, determined using the simple equation Δ_R_ = *R_i_* — *R_f_* (Figure S10B). The change in Δ_*R*_ was determined for each LNP formulation, at each temperature and in each excipient solution as summarized in Figure 1. Looking at the Δ_R_ alone, (+)LNPs appear to be the most stable LNP in every dispersant measured. (-)LNPs are the least stable. An issue that arose was that in some cases of LNP and dispersant combination, the ratio value for the LNP was not greatly different from the Cy5-siRNA in solution (Figure S10B) – suggesting that the buffer changes lead to a different conformation of the LNP during formation. Furthermore, (-)LNPs encapsulating negatively charged siRNA might experience repulsion effects, thus leading them to be less stable than (+)LNPs. To provide a more holistic view, Δ_R_ was plotted against initial ratio and the marker sizes used to indicate the temperature stress (Error! Reference source not found.). The plots allow us to visualize a region of stability in the upper left-hand corner of each plot, with larger shifts in initial ratio and lower Δ_R_ at increasing stress temperature easily observed for LNPs in 5 % mannitol and 5 % lactose, while PBS and 5 % trehalose show higher Δ_R_ values trending higher as the stress temperatures increases and lower initial ratios. Moreover, applying a temperature of up to 72 °C seemed not to have an influence of the LNPs stability in 5% lactose and 5% mannitol solutions (m/V). In the previous studies by Freitas and Müller, solid lipid nanoparticles were successfully spray-dried at outlet temperatures of 50-60 °C using mannitol, trehalose or lactose as the excipient solution. However, the particles were not carrying any cargo, and no *in vitro* or *in vivo* work was performed. [47]

Of the different excipient solutions tested, 5% mannitol and 5% lactose solutions were most effective at stabilizing the LNPs at the temperatures tested. Comparing these two excipient solutions more closely, the 5% lactose solution stabilized the different LNPs more efficiently at chosen temperatures. Furthermore, the Δ ratio within each LNP formulation remained lower than in the LNPs prepared in 5% mannitol solution. Hence, it seems favorable to use 5% lactose as an ideal excipient solution for spray drying of LNP formulations. In regards to the LNP carriers, the thermal stressing of LNPs up to 72 °C did not lead to any reduction of stability. RNA stability was not assessed, however. In conclusion, the highest possible temperatures, 62 °C and 72 °C, were selected for spray dry all LNP formulations in 5% lactose solution (m/V).

### 3.2 Characterization of spray dried LNPs

#### 3.2.1 Losses during spray drying

**Figure 3:**
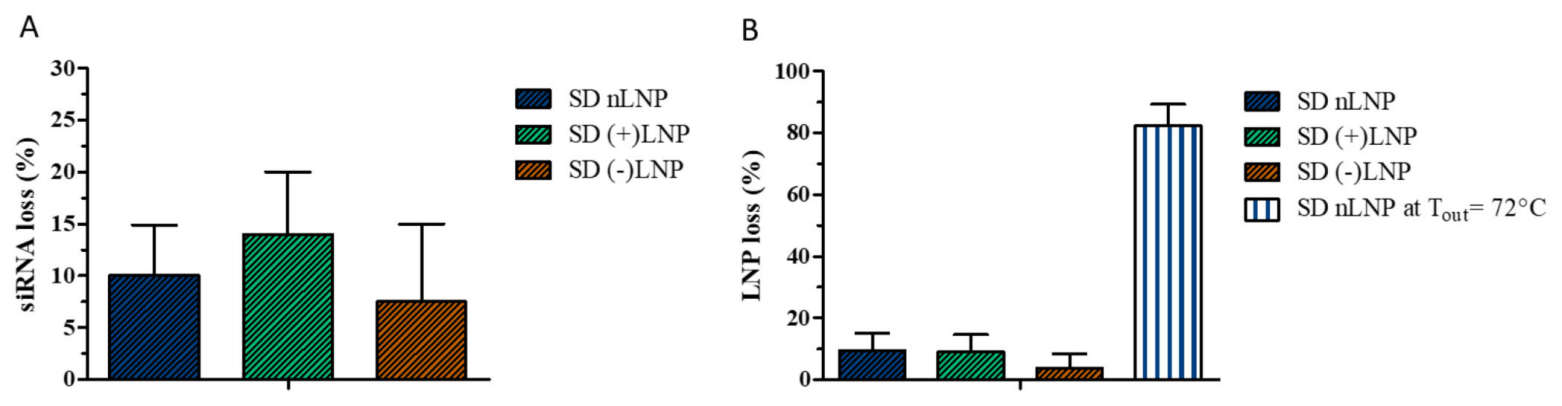
Quantification of the losses of A) siRNA and B) cholesterol after spray drying of LNPs in 5% lactose solution (m/V) at an outlet temperatures of 62°C. A nLNP loss at outlet temperature of 72°C is shown in B). Each bar shown as mean ± standard deviation, n=3. SD stands for spray-dried.

Spray drying of LNPs was performed in a Büchi B-290 spray drying tower applying inlet temperatures of 100 °C and 120 °C resulting in outlet temperatures of 62 °C and 72 °C, respectively. In order to ascertain whether the spray drying process results in LNP- and subsequently siRNA losses, the respective amounts were measured before and after spray drying. When spray dried at 62 °C outlet temperature, none of the LNP formulation showed high siRNA or LNP losses of more than 30%. The siRNA losses were shown to be 7.5 – 14.0% (Figure 3A). The cholesterol detection assay resulted in 3.85 – 9.54% LNP losses (Figure 3B). There were no significant differences between the lipid and the siRNA losses observed between the three LNP formulations. However, even though the dual emission fluorescence-based stability measurements were performed at the outlet temperature of 72 °C (T_in_ = 120 °C), this temperature led during the spray drying process to visual destruction of LNP samples. Only a very low amount of powder was collected showing streaks in the collection vial indicating that the LNP formulation had started to melt. Analysis of the small amount of collected powder showed high cholesterol losses over 80% (Figure 3B). Accordingly, the maximum temperature suitable for spray drying LNPs was set at an inlet temperature of 100 °C, resulting in an outlet temperature of 62 ± 2 °C.

#### 3.2.2 Physicochemical properties of spray dried LNPs

**Figure 4:**
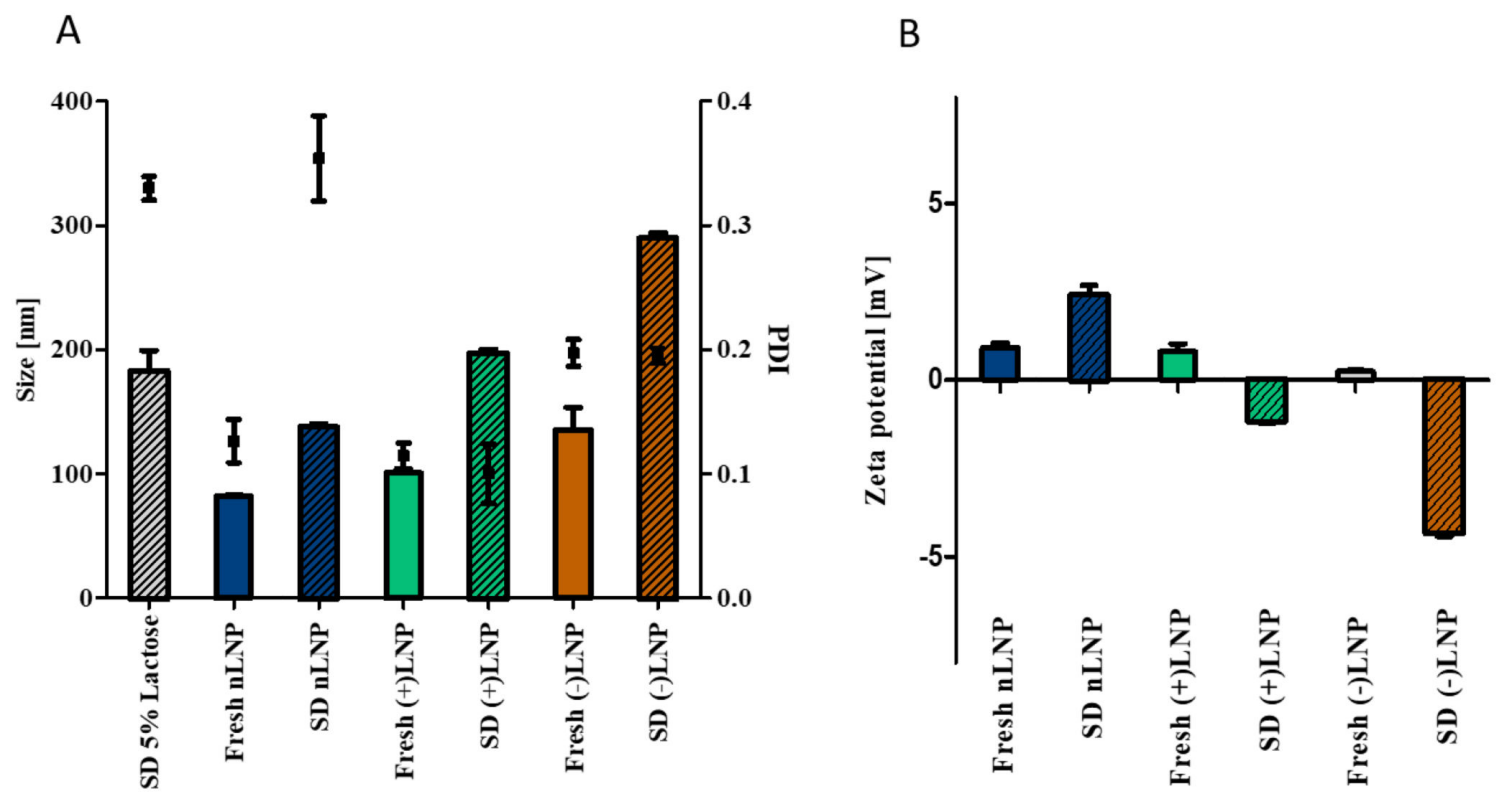
A) DLS measurements of freshly prepared (full colored bars) and redispersed (shaded colored bars) LNPs. PDI is indicated by black squares. LNP formulations with neutral, positive or negative charge and spray dried (SD) 5% lactose were redispersed in HPW after spray drying at 62°C outlet temperature and compared to freshly prepared LNPs in 5% lactose solution. B) Zeta potential measurements of fresh and spray dried LNPs in 5% lactose solution via LDA. Mean ± standard deviation, n=3.

Besides heat, spray drying exerts shear forces on LNP-siRNA systems and could melt, disassemble, destroy or merge LNPs. Therefore, DLS measurements were performed before and after spray drying to visualize any possible effects. Subsequently, spray dried microparticles, having the LNPs embedded, were dissolved in HPW for LNP redispersion to mimic impaction and matrix excipient dissolution in the lungs. As demonstrated in Figure 4, Z-average values of freshly LNPs prepared in 5% lactose (m/V) and redispersed LNPs from spray-dried powders did not show any statistical differences. Also, differences in PDI were not observed except for redispersed nLNPs. Here, the PDI increased slightly by keeping the sizes similar to before spray drying. However, we recognized higher PDI values which may be explained to some extent by sugar monomers that change the size distribution of the particles. It was shown by Weinbuch et al. that monomers of sugar and sugar alcohol are detected by DLS in highly concentrated solutions. [48] Furthermore, by measuring the size of redispersed spray dried 5% lactose solution, without any LNP cargo, we detected particle structures at 200 nm size and a PDI of over 0.3. It is expected that the sugar matrix does not instantly dissolve. By diluting the obtained lactose solution further, the count rate dropped and no significant peaks were detected (data not shown). Furthermore, a PEG-DMG loss from the LNP formulation could potentially explain the increased PDI and zeta potential changes. In summary, LNP size and distribution were not affected by spray drying and remained comparable to the freshly prepared samples. The zeta potential of LNPs, on the other hand, is dictated by their helper lipid. Neutral DSPC, positively charged DOTAP and negatively charged DSPG were implemented into the LNP structure to facilitate different characteristics. However, by spray drying LNPs in 5% lactose solution (m/V), the zeta potential of positively and negatively charged LNPs was reduced. It was therefore investigated in the following experiments whether the decreased zeta potentials changed *in vitro* cellular uptake.

**Figure 5:**
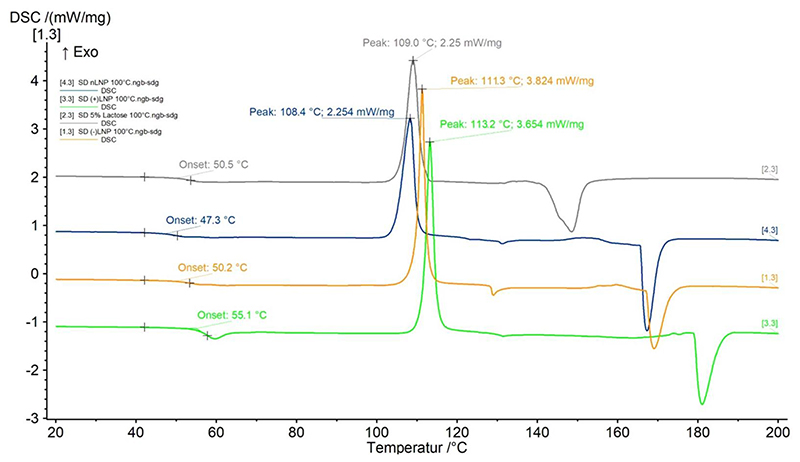
DSC measurements of lactose formulations spray dried at an outlet temperature of 62°C: 1.3) SD (-)LNP (brown), 2.3) SD 5% lactose (grey), 3.3) SD (+)LNP (green), 4.3) SD nLNP (blue).

As seen in Table 1, the average amount collected for all LNP formulations was around 65% yield at a maximum of 250 mg spray dried powder. This is in line with the expectations from a Büchi B-290 spray dryer which is stated to achieve a yield of about 70% [49] and is ranked at the upper end of collected yield in comparison to literature values. [50] Another important parameter that needs to be tested is the residual moisture. Spray dried powders should show low residual moisture levels in order to allow for storage stability. Although it was discussed above that residual moisture may act as a plasticizer stabilizing LNPs during the spray drying process, it could nonetheless cause microparticle aggregation lead to microbial growth and RNase contamination. Therefore, the moisture content of all formulations was measured by Karl Fischer titration. The results show that for all LNP formulations, independent of the charge of the LNP and the drying temperature, the residual moisture levels were 3.5 – 4%. Interestingly, spray drying 5% lactose solution without any LNPs showed a slightly higher residual moisture level of 4.9%. However, the differences are not significant and are in line within previously reported literature values. [44] Lactose often solidifies upon spray drying into an amorphous state. This was detected by DSC of spray dried 5% lactose solution in comparison to all other spray dried LNP formulations (Figure 5). In addition to the hygroscopic nature of lactose, the reason for the formation of amorphous structures is the fast drying step which does not provide sufficient time for lactose molecules to arrange into an ordered structure with subsequent crystal nucleation and growth. This explanation is supported by the molecule’s crystallization temperature of around 110 °C and melting points ranging from 150 °C to 180 °C. [51] All of the lactose formulations showed glass transitions at temperatures between 47 °C and 56 °C corresponding to their residual moisture content (Table 1). [44, 52, 53] This temperature (Tg) is important for stability predictions during storage as amorphous solid forms are thermodynamically unstable and tend to crystallize if stored close to or above Tg. [54] The amorphous state of the formulation is favorable for LNP preservation. Therefore, when storing these products at 4 °C or room temperature for a longer period of time, high Tg values are necessary. The final Tg of a formulation, however, is closely linked to the water content: the higher the residual moisture the lower Tg. Also, with a lower residual moisture content degradation processes are less likely to occur. [55] It is therefore of interest to further decrease the amount of residual moisture in lactose formulations to avoid nucleation and degradation processes over time and in order to maintain the amorphous state of the formulation.

**Figure 6:**
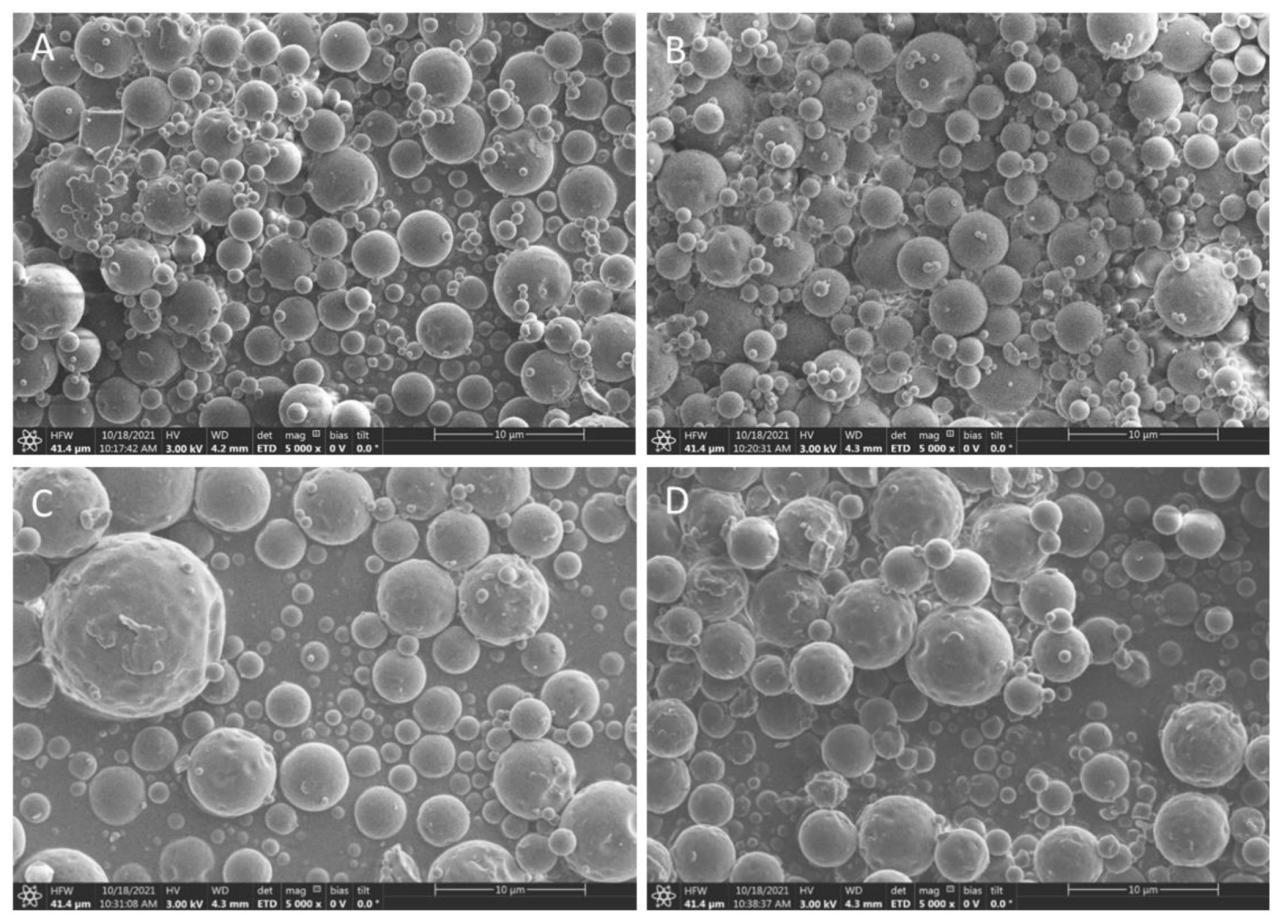
SEM pictures of spray dried A) 5% lactose solution, B) nLNP formulation, C) (+)LNP formulation and D) (-)LNP formulation. All samples were spray dried in 5% lactose solution at 62°C outlet temperature.

When administration of spray dried powders application is envisioned via the pulmonary route, their morphology and particle sizes need to be examined. Aerodynamic sizes of 1 – 5 μm are considered ideal for inhalation because more than 50% of particles of an aerodynamic size of 3 μm deposit in the alveolar region. If the particle sizes are smaller than 3 μm, an 80% chance of reaching the lower airways and a 50-60% chance of deposition in the alveoli is given. [56–58] Therefore, the optimal aerodynamic size for deep lung deposition after pulmonary delivery is around 3 μm. For local effects, aerodynamic particles sizes around 5-7 μm can as well be acceptable. To determine whether the spray-dried particles in this study fit these criteria, they were imaged using SEM. The geometric median diameters (GMD) which reveal the actual visual particle size. As seen in Figure 6, spray drying of all LNP formulations with 5% lactose solution resulted in smooth round microparticles of sizes below 10 μm. In Figure 6A, spray dried 5% lactose, and 6B, spray dried nLNPs, showed a GMD size range from particles of 2 – 7 μm, whereas, (+)LNPs and (-)LNPs were slightly larger with 3 – 9 μm (Figure 6C and D). For porous materials, geometric sizes commonly exceed the aerodynamic diameter [59], which was therefore examined experimentally. Furthermore, the residual moisture has a direct impact on the particles’ size with higher water content resulting in more particle aggregation and bigger GMD values accordingly.

#### 3.2.3 Aerodynamic performance of spray dried LNPs

The aerodynamic performance of each spray dried LNP formulation was measured using an NGI. The mass median aerodynamic diameters (MMAD) of all powders present sizes of 2.85 – 2.9 μm with standard deviations of 0.07 – 0.42 μm. These results position the spray dried LNP formulations at aerodynamic sizes of about 3 μm which was discussed as the optimal particle size range for lower respiratory delivery targeting a wide field of pulmonary diseases such as COPD, or respiratory viruses, e.g., influenza or SARS-CoV-2. This is underlined by the fact that the geometric standard deviation (GSD) remains low at 2 μm. Reaching a fine particle fraction (FPF, defined as particles under 5 μm in diameter) of almost 30% is a very good value that is in line with the geometric diameter results obtained from SEM pictures. The FPF can be improved by reducing the residual moisture of spray dried LNPs to narrow down the size distribution, GSD, of spray dried powders and avoid particle agglomerates. This can be achieved by drying the spray dried powder in a subsequent drying step at a lower inlet temperature to reduce the heat stress on the product. The temperature should not be increased since the spray drying temperature was already set to an upper threshold value keeping the LNPs integrity. The fine particle dose (FPD) expresses the FPF value in an absolute mass. Therefore, 0.74 μg siRNA were delivered at sizes below 5 μm out of 2.6 μg siRNA applied, known as dose delivered (DD). The overall recovery rate ranged from 71.1% to 86.8%. Some losses could have been caused by an insufficient clearance of capsules using the Handihaler device. Furthermore, depending on the surface charge of the LNPs, a difference in electrostatic charge was noticeable that could have led to higher adhesion of powders to the walls of capsules, induction port and pre-separator. The conclusion can be drawn that for all the different powders, regardless of their different LNP cargo, optimal microparticulate characteristics for pulmonary application were achieved.

### 3.3 Mucus penetration of spray dried LNPs

**Figure 7:**
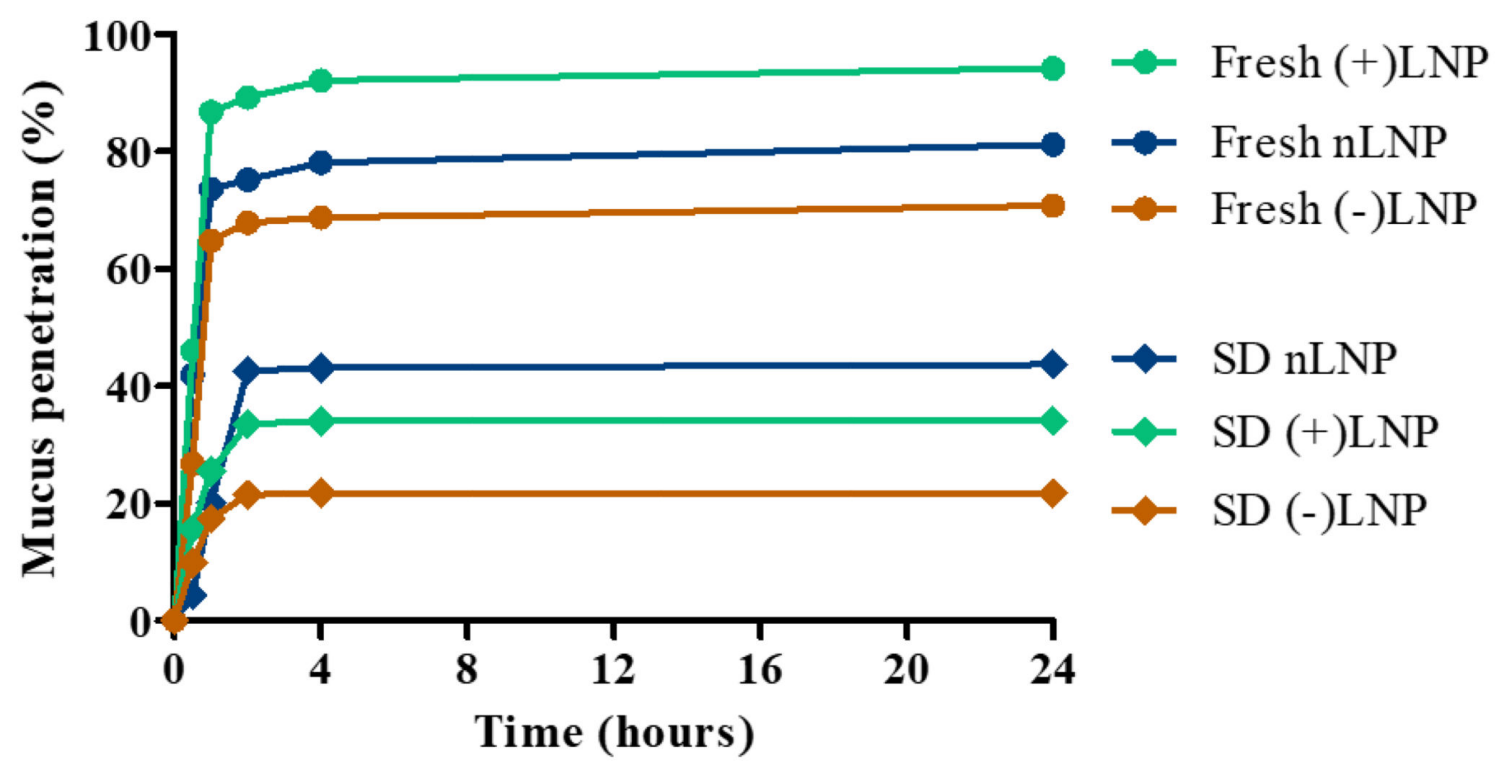
Mucus penetration assay of fresh LNPs vs spray dried (SD) LNPs. The time points were chosen at 0h, 0.5h, 1h, 2h, 4h and 24h.

For efficient delivery of spray dried powder to the lungs a few hurdles need to be overcome. First, the nano-in-microparticles need to be redispersed in lung fluid quick enough to dissociate into nanoparticles. Second, the nanoparticles need to pass the lung fluid as quickly as possible before the fluid is renewed and all particles are washed away. To mimic this scenario, we performed a mucus penetration study, in which we compared the penetration ability of freshly prepared LNPs in 5% lactose solution with spray dried LNPs redispersed in HPW. By taking into account the LNPs zeta potential which remained neutral for all LNP formulations in 5% lactose solution (m/V), LNPs are expected to pass the mucus layer without diffusion restrictions due to charge interactions. [60] As seen in Figure 7, 65-90% of the freshly prepared LNPs penetrate the mucus layer, whereas only 20-40% of the spray dried LNP penetrate the mucus. This higher amount of penetration for freshly prepared LNPs can be explained by referring to the sizes of redispersed LNPs which lets us assume that the sugar matrix was not fully dissolved resulting in bigger nanoparticle sizes. Moreover, the spray dried LNPs were still immobilized inside the sugar matrix not being able to pass the mucus layer as efficiently as freshly prepared LNPs. It is noteworthy, that the negatively charged, (-)LNPs, showed the least efficient mucus diffusion both as freshly prepared or spray dried particles. Comparing our results to the literature, a negatively charged particle should pass through the negatively charged mucus more efficiently because of charge repulsion. A positively charged particle would interact with the negative mucus charge and be hindered in diffusion. A neutral charged particle would pass the mucus layer but not as efficiently as the negative ones. [35, 60] Freshly prepared (+)LNPs show mucus penetration characteristics of 90% which contradicts the charge theory. However, low nanoparticle sizes and near to neutral zeta potential in 5% lactose solution can explain its efficient mucus penetration. Spray dried nLNPs showed the highest penetration at 40%. In conclusion, the spray dried LNPs are capable of penetrating a lung mucus layer in order to reach the lung cells to release the siRNA cargo even if to a lesser extent than their freshly prepared counterparts.

### 3.4 *In vitro* characterization of spray dried LNPs

**Figure 8:**
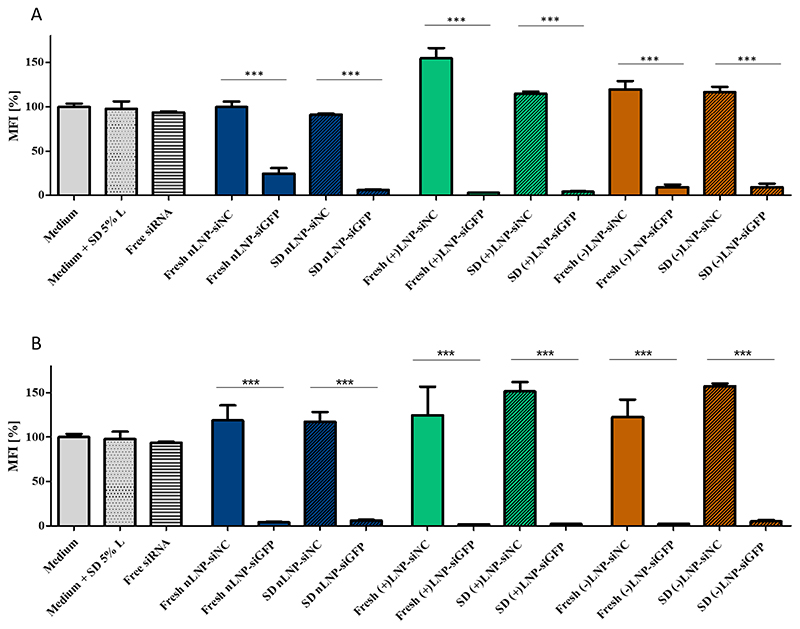
*In vitro* gene silencing effect of enhanced green fluorescent protein (eGFP) within a H1299-eGFP expressing cell line. Different siRNA concentrations were tested: A) 1 μg/mL, B) 10 μg/mL. Samples are plotted as follow: freshly prepared LNPs in full colored bars, spray dried LNPs in shaded colored bars. Bars show the mean fluorescent intensities (MFI) of the eGFP as a percentage relative to the untreated sample (grey, unpatterned bar).

For siRNA delivery it is fundamental to maintain the molecule's bioactivity throughout the spray drying and storage process. Since the outlet temperature of 62°C is not close to the degradation temperature of siRNAs and no severe losses of LNPs or siRNA were detected as abovementioned (Figure 3), the bioactivity of the LNPs was expected to be intact. Therefore, the gene silencing efficiency of spray dried LNPs (nLNP, (+)LNP and (-)LNP) of the enhanced green fluorescence protein expressing H1299 (H1299-GFP) cells were tested. All LNPs had an siRNA against GFP (siGFP) encapsulated. Furthermore, for comparison, another set of LNPs was prepared with scrambled, negative-control siRNA (siNC). Freshly prepared LNPs were dispersed in 5% lactose solution (m/V), whereas spray dried LNPs were redispersed in HPW. The specific amount of fresh LNPs and spray dried LNPs was chosen to transfect the cells at an siRNA concentration of 1 μg/mL (55.7 nM siGFP/siNC) and 10 μg/mL (557 nM siGFP/siNC), respectively. As other controls, spray dried 5% lactose solution was redispersed in HPW and free siRNA in the same amount was added to the cells. As seen in Figure 8A and 8B all LNPs having siGFP encapsulated show a highly significant GFP downregulation effect on the protein level. The downregulation ranged from approximately 80% for freshly prepared nLNPs at 1 μg/mL to > 95% downregulation of fresh (+)LNPs at 10 μg/mL. All control values showed no gene knockdown effect. Hence, the gene silencing efficiency is RNAi mediated and a result of the complementary siRNA sequence. At 1 μg siRNA/mL, Figure 8A, freshly prepared nLNPs performed least efficiently of all LNP formulations with 80% downregulation, in comparison to 90% knockdown of spray dried nLNPs. This difference can be explained by the charge difference of freshly prepared LNPs in comparison to redispersed spray dried LNPs. More positively charged nanoparticles show a greater interaction with the cell membrane through attractive electrostatic interactions with negatively charged phospholipids or membrane proteins, and subsequently lead to a higher cell uptake. [61, 62] Therefore, a higher eGFP knockdown can be the result of the aforementioned with an increased amount of siRNA entering the cells. (-)LNPs showed 90% gene silencing and were outperformed with >95% performance by (+)LNPs. By increasing the siRNA amount 10-fold, the gene silencing effect increased throughout all LNP formulations to values > 90% (Figure 8B). The negative control, scrambled siRNA values remained above 100% for each LNP formulation reflecting a highly significant gene silencing effect. To compare the LNP formulations against each other, (+)LNPs outperformed the other two formulations, even at lower siRNA concentrations. This observation can be explained by (+)LNPs entering the cell more easily and releasing higher amounts of siRNA from the endosome. [63] The 5% lactose solution should not interfere with the charges since the cells were cultivated in 400 μL cell medium and 100 μL of redispersed LNPs. This dilution could have enhanced the dissolution of LNPs out of the sugar matrix. Noteworthy, the gene silencing effects do not differ between freshly prepared and spray dried LNPs, and a knockdown on protein level was successfully achieved. This underlines the excellent characteristics of the LNPs which did not change after the spray drying process. To compare our results to previous studies, Jensen et al. spray dried DOTAP modified PLGA nanoparticles loaded with siRNA in mannitol at an outlet temperature of 30°C to obtain hybrid dry powder formulations. Performing eGFP knockdown experiments in H1299-GFP cells resulted in a maximum silencing of 73%. [64] This knockdown result was one of the highest found in literature but not close to our outcome for the hybrid system spray dried at low temperatures. In another study, Karve et al. spray dried mRNA embedded hybrid nanoparticles at an outlet temperature of 46-50°C. Neither *in vitro*, nor *in vivo work* was performed and the attempt to spray dry lipid particles without polymer pre-encapsulation resulted in a recovery rate of 1-2%. [65] The temperature increase came in cost of the recovery rate. Adding a polymer to stabilize the system helped the authors to create a more feasible system. This confirms the difficulty of spray drying lipid nanoparticles at the highest possible temperatures while keeping the composition and bioactivity of the cargo. To the best of our knowledge, we are the first to report a successful eGFP knockdown of over 95% after spray drying of Onpattro®-derived LNPs at an outlet temperature of 62°C. Neither different spray dried lipid based nanoparticle systems, nor hybrid or polymeric nanoparticulate systems have shown a similar eGFP *in vitro* activity.

**Figure 9:**
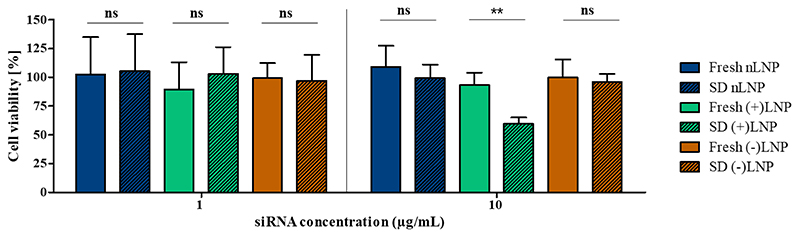
*In vitro* cytotoxicity evaluation via a MTT assay in H1299-eGFP cells. The siRNA concentrations were set at 1 μg/mL and 10 ug/mL for all samples. Samples are plotted as follow: freshly prepared LNPs in full colored bars, spray dried LNPs in shaded colored bars.

To exclude any toxic effects originated from LNP formulations, a cytotoxicity evaluation via an MTT assay was performed. All samples show no toxic effects and results are not statistically significantly different for freshly prepared LNPs vs. spray dried LNPs, apart from (+)LNP at a siRNA concentration of 10 μg/mL (Figure 9). This cytotoxic effect of about 35% could result from combining the positive LNP charge with a high amount of sugar being added to the cells. As discussed in literature and shown for many nanoparticulate systems, high positive charges cause increased toxicity to the cells. [62, 66] By increasing the siRNA amount by 10 fold, we also increased the (+)LNP amount. This could cause an increased impact of positive charge to the cell membrane leading to cell disruption and cell death. Furthermore, an increasing amount of sugar can cause increased osmotic effects on the cells, inevitably, resulting in the same outcome. However, all other LNP formulations, redispersed after spray drying, do not show any cytotoxic effects. Hence, the main cytotoxic reason results from its positive charge. In summary, it was determined that a siRNA concentration of no higher than 10 μg/mL was necessary for achieving adequate gene knockdown, and higher concentrations were likely to increase the risk of cytotoxicity.

### 3.5 *Ex vivo* activity of spray dried LNPs in human precision-cut lung slices (hPCLS)

Human PCLS represent complex *ex vivo* 3D tissue culture models closely mimicking the anatomy and physiology of the lungs by maintaining the structure and cellular diversity. Furthermore, by closing the translational gap between *in vitro* and *in vivo* models, PCLS enable the study of respiratory diseases such as allergic asthma [67], COPD [68, 69], IPF [70] and viral infections [71] and can act as a more sophisticated nucleic acid delivery model to the lungs. [72–74]

**Figure 10:**
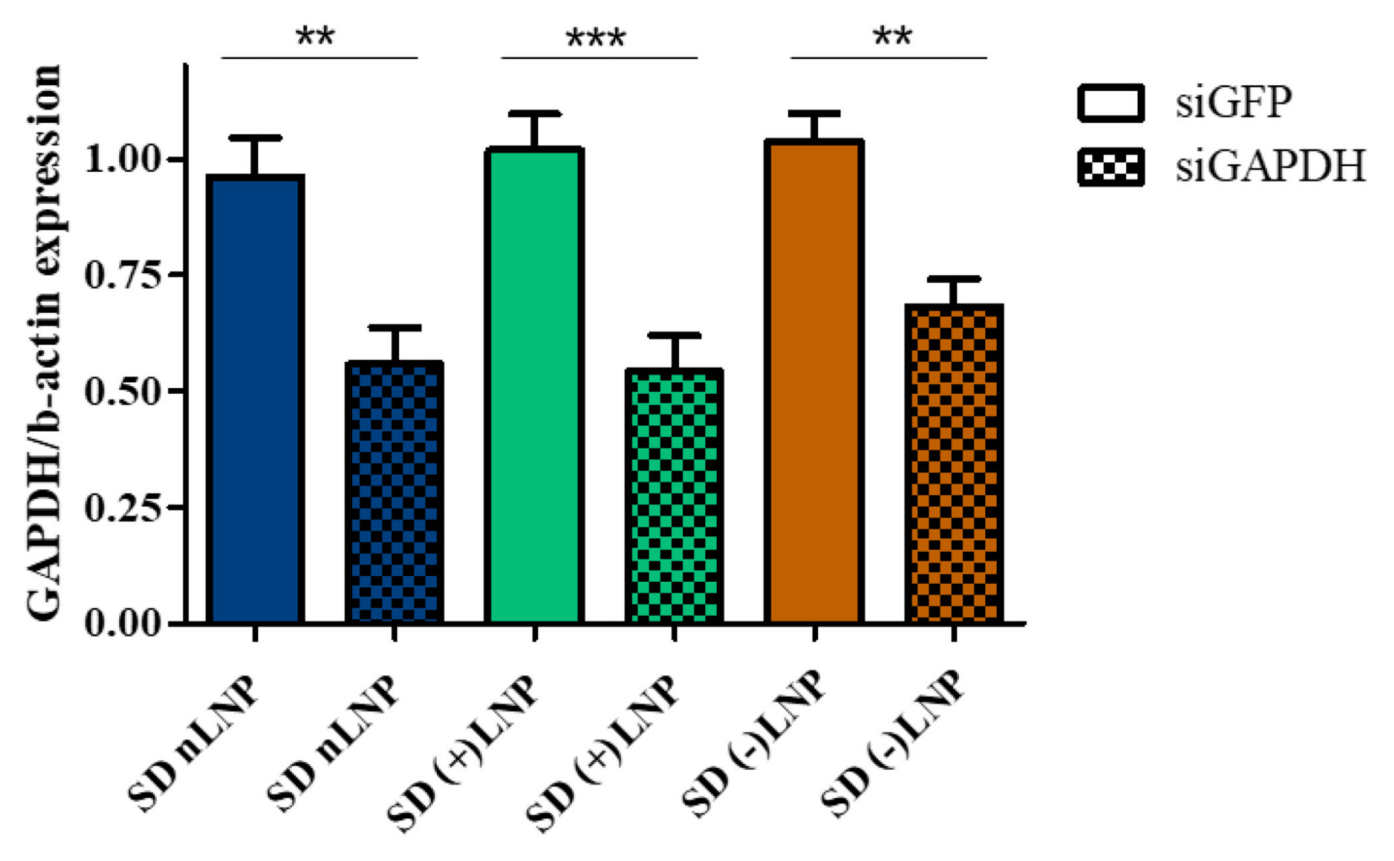
*Ex vivo* knockdown of house-keeping gene GAPDH. Human precision cut lung slices (hPCLS) were transfected at 10 μg siRNA / mL with spray dried LNPs encapsulating either siGAPDH or siGFP. All values were expressed as a percentage in comparison to the baseline values of samples treated with LNP-siGFP. Mean ± standard deviation, n=3.

Following the investigation of successful gene silencing on the protein level using spray dried LNPs, human precision cut lung slices (hPCLS) of individual donors were used to evaluate the gene knockdown efficiency of LNPs on the mRNA level. Human PCLS were transfected at siRNA concentrations of 10 μg/mL using redispersed spray dried LNP formulations targeting the house keeping gene GAPDH (siGAPDH). As a reference control, spray dried LNP-siGFP formulations were used to rule out any off-target effects. Untreated control hPCLS were treated with medium and 5% lactose solution only. Figure 10 shows the normalization of GAPDH mRNA by the mRNA levels of β-actin and all values being normalized against LNP-siGFP values. The hPCLS treated with spray dried LNPs-siGAPDH showed significant reduction of GAPDH expression for each LNP formulation tested. The knockdown results ranged from 35% in (-)LNPs to 50% in (+)LNPs. The nLNP formulation showed a silencing efficiency of 45%. Figure S11 underlines that no statistically significant inflammatory effects were observed after transfection of hPCLS with different LNPs based on the level of twelve different proinflammatory cytokines. As stated in the *in vitro* protein silencing experiment, the (+)LNPs outperformed the other LNP formulations demonstrating their drug delivery potential for pulmonary administration. The overall performance of LNPs being able to reduce the mRNA level up to 50% underlines the LNPs’ preservation of bioactivity and transfection efficiency after spray drying. Most importantly, the LNP formulations did mediate a level of *ex vivo* gene knockdown that has not been observed before with spray dried LNP delivery systems targeting the lungs. In comparison, Ruigrok et al. reported approximately 50% gene silencing of GAPDH in murine PCLS using non-spray dried Accell siRNA not using a nanocarrier delivery system [74]. As discussed in the beginning, naked siRNAs cannot cross the cell membrane sufficiently and need to be protected from heat stress during the spray drying process, hence, a galenic packaging of the cargo is necessary. Moreover, pulmonary administration is most commonly achieved via nebulization or dry powder inhalation. Furthermore, the LNPs’ stability to retain the siRNA after spray drying highlight robustness and manifoldness of LNP formulations for pulmonary application systems. Those aspects underline the complexity of spray drying of LNP-siRNA formulations for pulmonary application.Therefore, our results of 50% *ex vivo* gene silencing of GAPDH RNA levels emphasize the relevancy of spray dried LNPs as a promising therapy for the treatment of respiratory diseases such as asthma, COPD, lung cancer, cystic fibrosis or viral infections.

## 4 Conclusion

In this study, we established a spray drying setup that allows RNA-loaded lipid nanoparticle systems to be spray dried at the highest possible temperature while retaining the LNP structure, cargo integrity and maintaining bioactivity and gene silencing efficiency. The detection of thermal stability of LNPs using the dual emission fluorescence-based method enabled a prescreening of different LNP formulations in different excipient solutions and at different temperatures. It was beneficial to test LNP stabilities near the lipids’ phase transition temperatures to understand whether a spray drying process would damage the LNP composition or lead to cargo leakage. In addition, it enabled measurements of low sample volume and wide temperature ranges to simulate heat stress on the individual LNP systems during spray drying. These results led to a preselection of spray drying parameters including the best suitable excipient solution. Therefore, spray drying was performed in 5% lactose solution (m/V) in combination with a maximum spray drying inlet temperature of 100°C (62°C outlet temperature). Quantification measurements of spray dried LNPs resulted in low losses underlining the preservation of siRNA and LNPs. The spray dried microparticles demonstrate optimal physicochemical and aerodynamic properties for pulmonary administration to the lower respiratory tract. Spray dried LNP formulations were able to successfully pass an artificial mucus layer similarly found in human lungs. Efficient gene silencing on the protein level was achieved *in vitro* in an adenocarcinoma cell line showing very good cellular compatibility. Spray dried LNPs efficiently silenced the house keeping gene GAPDH in *ex vivo* human lung tissues. In conclusion, our research confirms the successful spray drying of LNP-siRNA formulations to create a novel siRNA-based therapy to target respiratory diseases such as lung cancer, asthma, COPD, cystic fibrosis and viral infections.

## Supplementary Material

Graphical Abstract

Supplementary Material

## Figures and Tables

**Figure 2:**
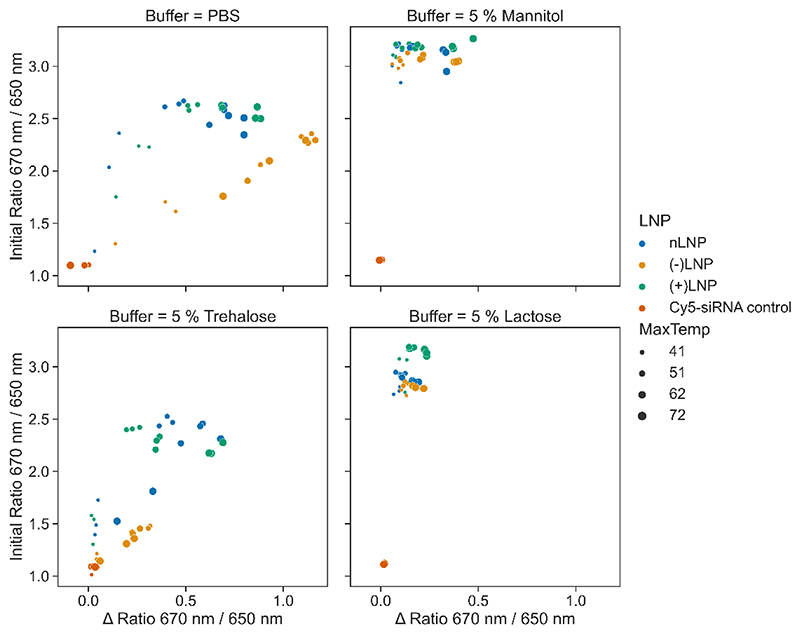
Stability plot shows stability of LNPs in each excipient buffer condition. The ΔR is plotted against initial ratio in each buffer condition, with the color indicating LNP and the size of marker stating the temperature the LNPs were stressed at. The top left quadrants represents the most stable environments.

**Table 1:** Residual moisture recovered spray-dried mass and yield of LNPs spray dried with 5% lactose (w/V) at an outlet temperature of 62°C.

Name	SD mass (mg)	SD yield (%)	Residual moisture (%)
SD 5% Lactose	192.06 ± 3.44	76.83 ± 1.37	4.90 ± 0.01
SD nLNP	160.89 ± 5.69	64.36 ± 2.28	4.01 ± 0.79
SD (+)LNP	164.15 ± 4.35	65.66 ± 1.74	4.12 ± 0.53
SD (-)LNP	164.69 ± 3.55	65.87 ± 1.42	3.56 ± 0.40

**Table 2:** Microparticle characteristics of spray dried LNP formulations in 5% lactose solution at an outlet temperature of 62 °C using a Next Generation Impactor (NGI). DD: dose delivered, FPD: fine particle dose, FPF: fine particle fraction, MMAD: mass median aerodynamic diameter, GSD: geometric standard deviation.

	nLNP	(±)LNP	(-)LNP
DD (μg)	2.37 ± 0.85	2.62 ± 0.31	2.66 ± 0.70
FPD (μg)	0.70 ± 0.27	0.74 ± 0.13	0.73 ± 0.10
FPF (%)	29.5 ± 0.60	28.1 ± 1.70	28.1 ± 3.80
MMAD (μm)	2.85 ± 0.07	2.85 ± 0.35	2.90 ± 0.42
GSD (μm)	1.96 ± 0.02	2.01 ± 0.05	2.01 ± 0.16
Recovery (%)	71.1 ± 28.1	82.9 ± 4.50	86.8 ± 17.3
