# Supplementary figures and images for: Spray drying siRNA-lipid nanoparticles for dry powder pulmonary delivery
Source: J Control Release. Author manuscript; Available in PMC 2022 Oct 13. (PMC7613708; doi:10.1016/j.jconrel.2022.09.021)

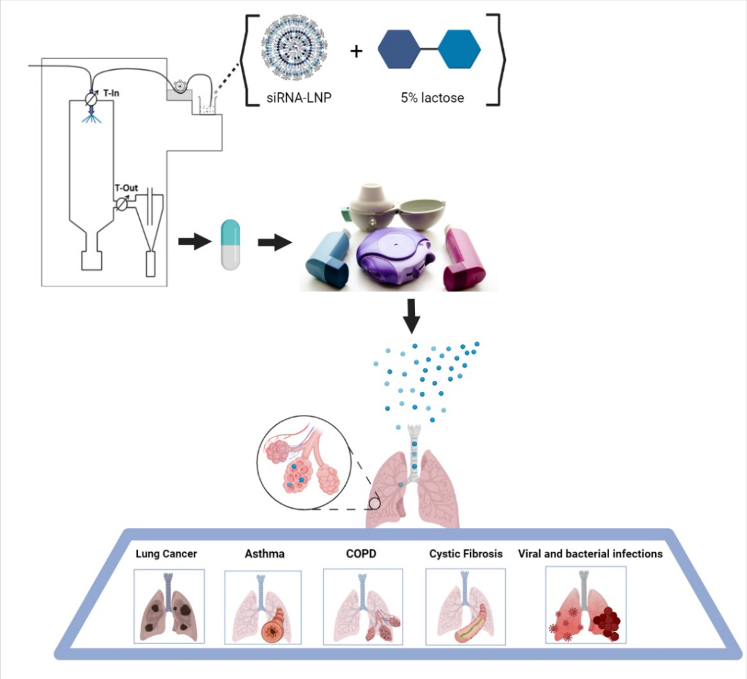

Supplement: Graphical Abstract [file EMS155326-supplement-Graphical_Abstract.png]
