# Supplementary material for: Spray drying siRNA-lipid nanoparticles for dry powder pulmonary delivery
Source: J Control Release. Author manuscript; Available in PMC 2022 Oct 13. (PMC7613708; doi:10.1016/j.jconrel.2022.09.021)
Supplement: Supplementary Material [file EMS155326-supplement-Supplementary_Material.docx]

**SUPPLEMENTARY MATERIAL**

**Table S1** Sequences of siRNAs used in the study. Nt = nucleotides; GFP = green fluorescence protein; NC = negative control; GAPDH = housekeeping gene GAPDH; A = Adenine; C = Cytosine; G = Guanine; U = Uracil; T = Thymine; p = phosphate residue; lower case bold letters = 2´-deoxyribonucleotides; capital letters = ribonucleotides; underlined capital letters = 2´-O-methylribonucleotides.

| Name | Sense strand (5’-3’) | Antisense strand (3’-5’) | Length (nt) | |
| --- | --- | --- | --- | --- |
|  |  |  | **Sense** | **Antisense** |
| siGFP | pACCCUGAAGUUCAUCUGCACCAC**cg** | ACUGGGACUUCAAGUAGACGUGGUGGC | **25** | **27** |
| siNC | pCGUUAAUCGCGUAUAAUACGCGU**at** | CAGCAAUUAGCGCAUAUUAUGCGCAUAp | **25** | **27** |
| siGAPDH | pGGUCGGAGUCAACGGAUUUGGUC**gt** | UUCCAGCCUCAGUUGCCUAAACCAGCA | **25** | **27** |


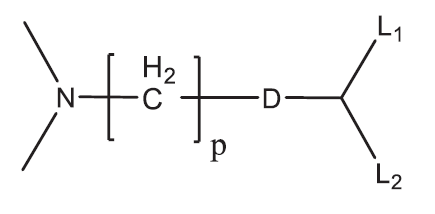


**Figure S1**Chemical structure of sulfur-containing analog of DLin-MC3-DMA.


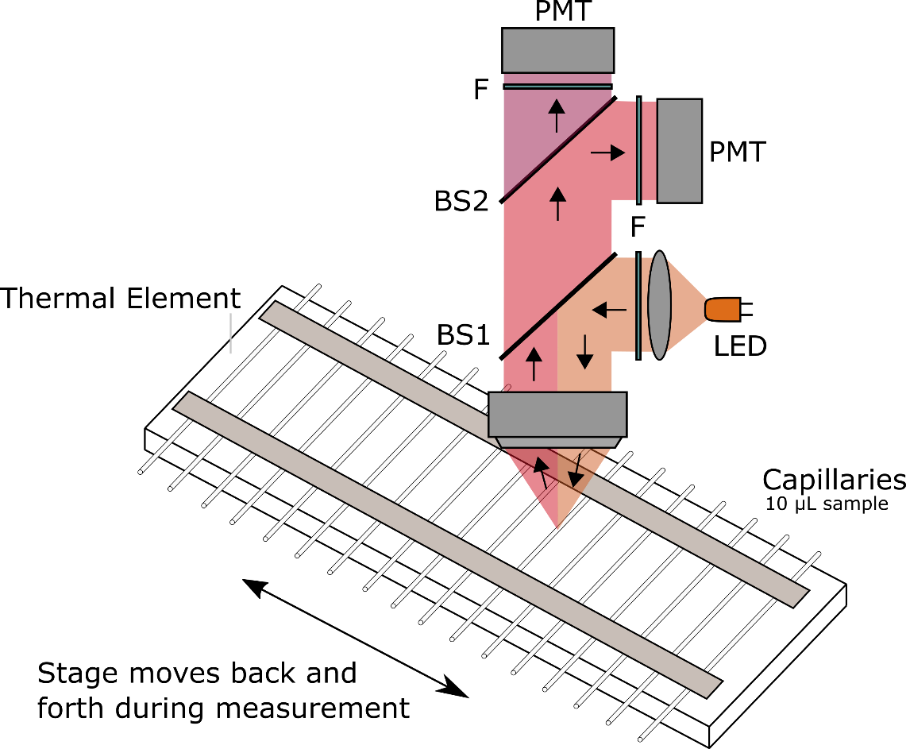


**Figure S2** Experimental epifluorescence setup. An amber LED produces excitation light that matches the secondary, shorter absorption peak of a suitable near-infrared fluorophore attached to siRNA. The light is reflected on a beam splitter (BS1) and excites fluorescence within a capillary. The red-shifted emission then passes BS1 and is divided by a second beam splitter (BS2) into a lower and higher wavelength component. Filters (F) further clean up the emission light before being collected in photo-multiplier tubes (PMT).


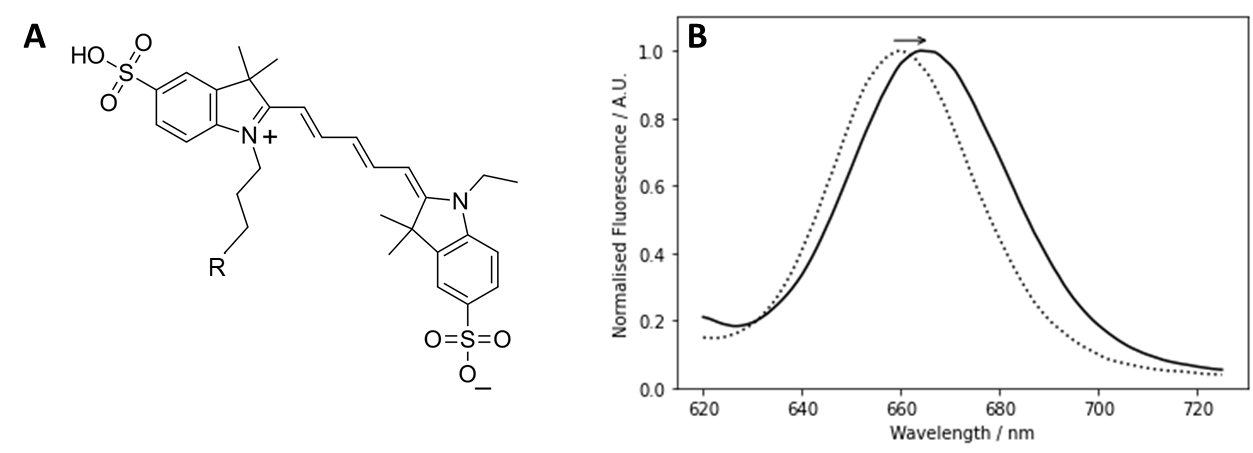


**Figure S3A)** Chemical structure of the Cyanine 5 dye, R group is attachment site to protein or nucleic acid. **B)** Cy5-siRNA (dashed line) displays a 6 nm bathochromic peak shift after encapsulation within an LNP (solid line). Cy5-siRNA was encapsulated within (-)LNP and diluted in PBS before excitation at 590 ± 20 nm, with emission recorded from 620 – 725 nm with a 10 nm band gap. Spectra were recorded in a temperature-controlled sample chamber at 20 °C.


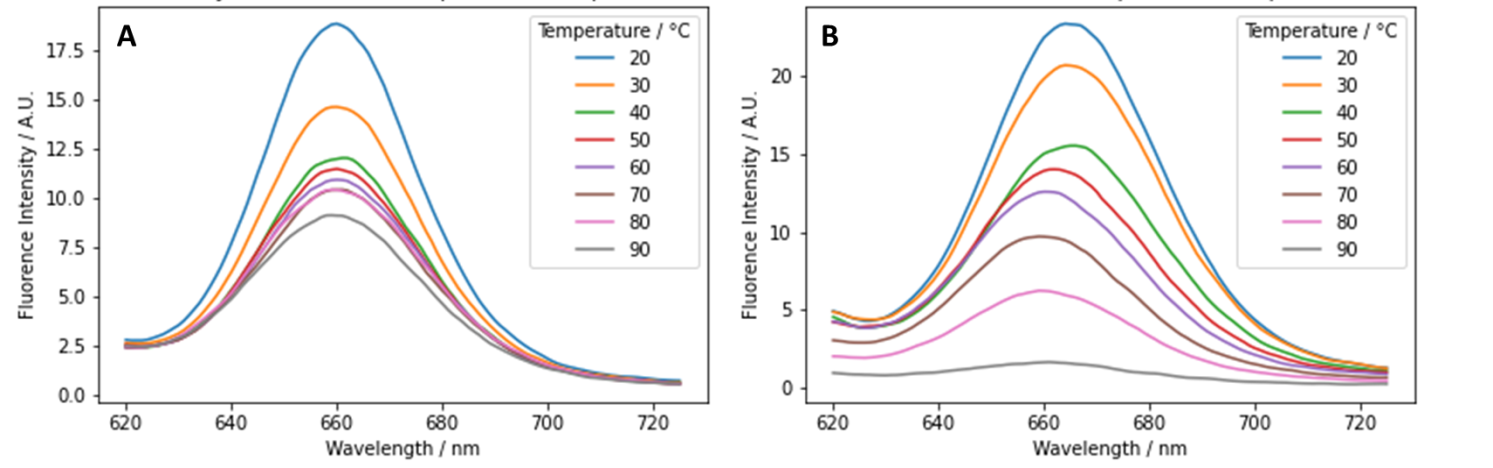


**Figure S4** Temperature Interval Fluorescence Emission Spectroscopy of **A)** Cy-siRNA and **B)** Cy-siRNA encapsulated in(-)LNP. Fluorescence emission scans were performed every 10 °C with a heating rate of 2 °C per min.


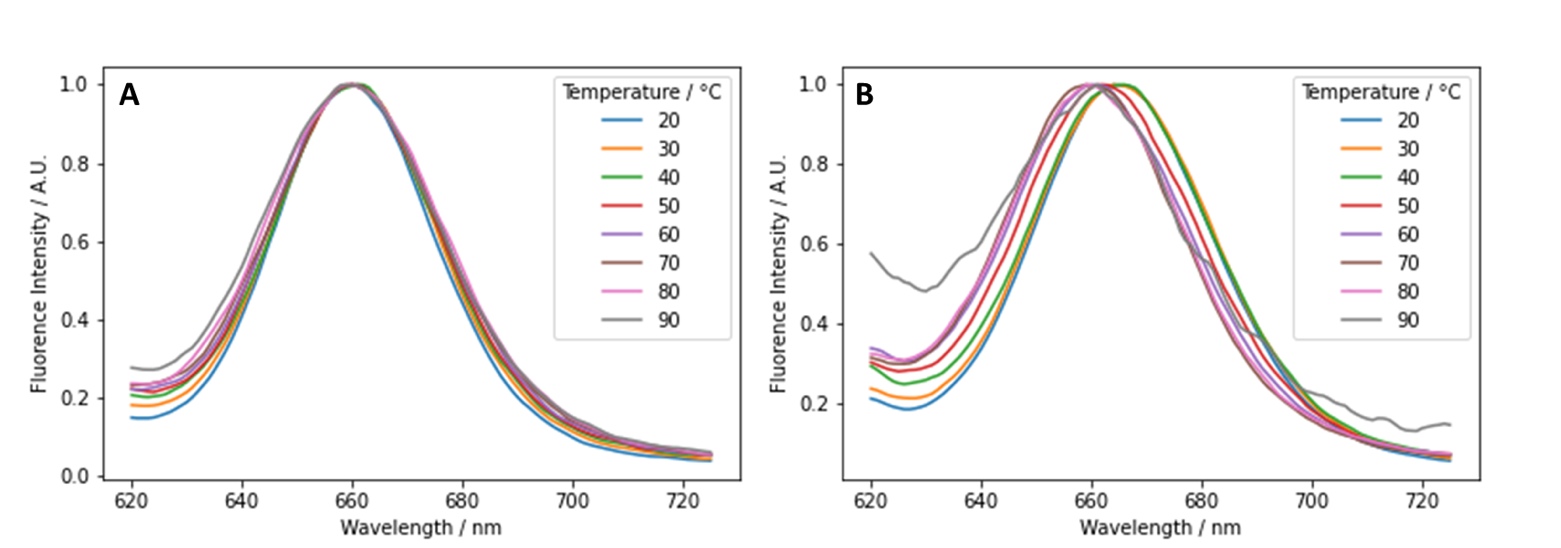


**Figure S5**Hypsochromic peak shift, normalized Spectra from Figure S3. Temperature Interval Fluorescence Emission Spectroscopy of **A)** Cy-siRNA and **B)** Cy-siRNA encapsulated in LNP5. Fluorescence emission scans were performed every 10 °C with a heating rate of 2 °C per min.


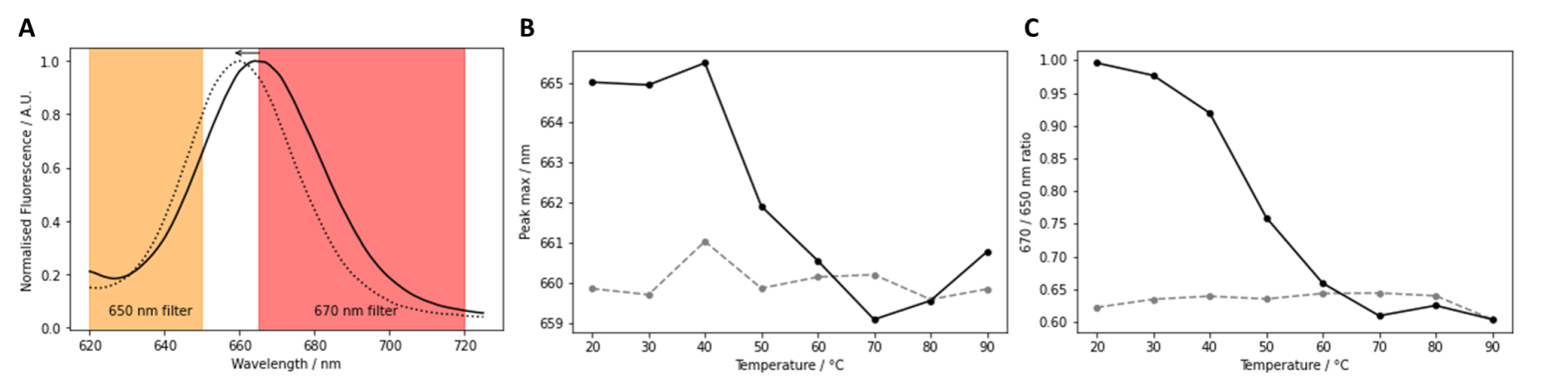


**Figure S6**Fluorescence basedmeasurements. **A**) Fluorescence emission spectra of Cy5-siRNA encapsulated within (-)LNP (black solid line) and Cy5-siRNA (dashed line) in PBS. Arrow indicates the shift in peak between the two samples, with coloured shading indicating the two spectra windows used for 650 nm and 670 nm ratio measurements. **B)** Peak shift and **C)** 670 nm / 650 nm ratio plotted against sample temperature of (-)LNP (black markers) and Cy5-siRNA (grey markers).


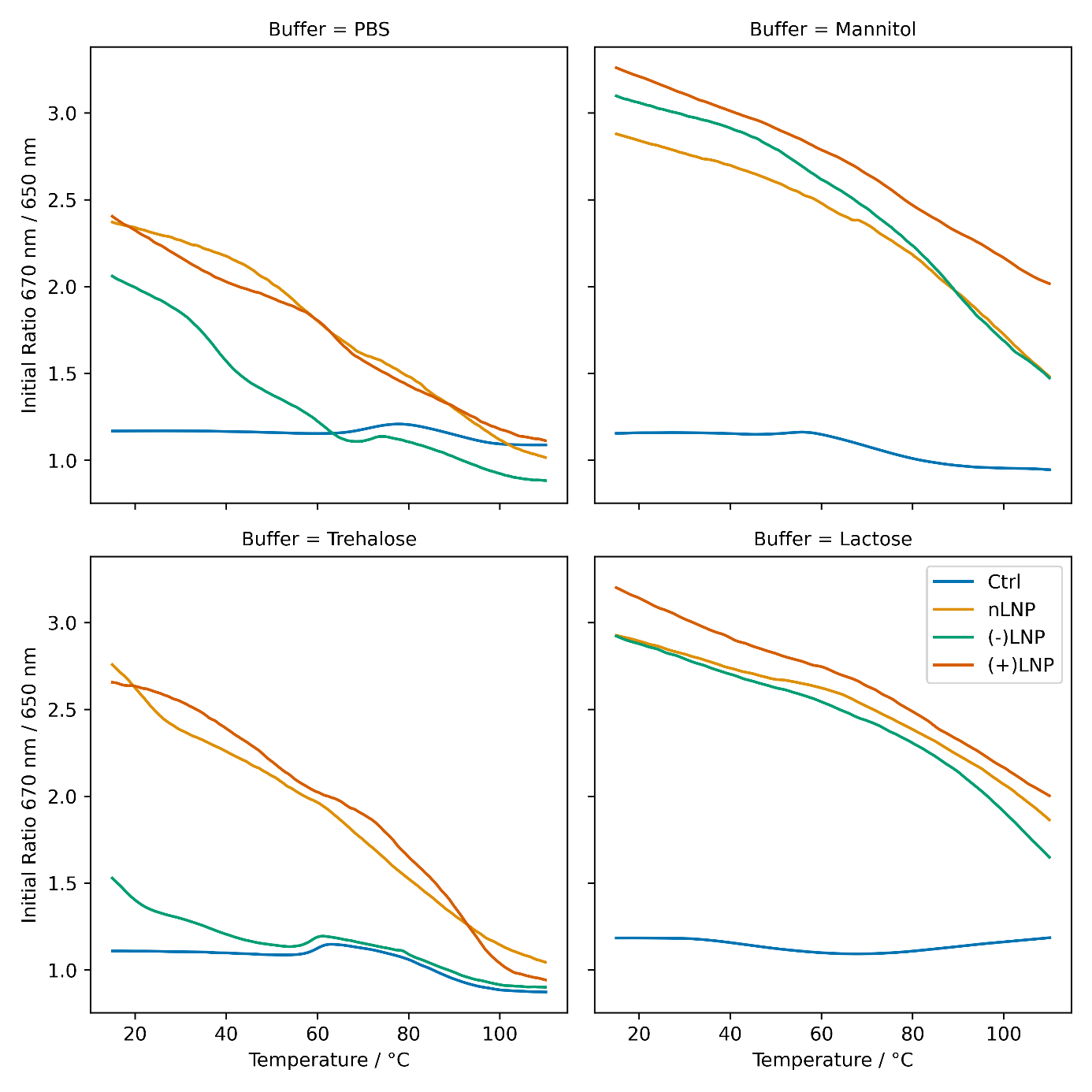


**Figure S7** 670 nm / 650 nm ratio of LNP samples in different excipient solutions (PBS, mannitol, trehalose and lactose) plotted against temperature of sample. Samples were loaded into high sensitivity capillaries and subjected to a temperature ramp rate of 1°C/min.


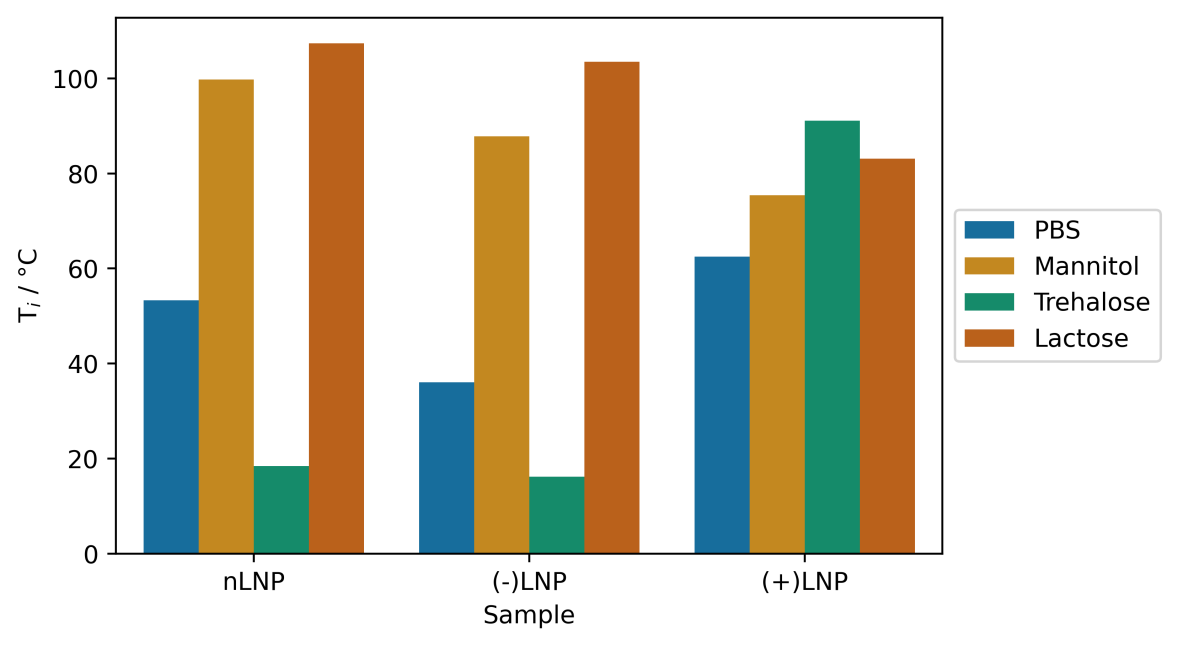


**Figure S8** Category plot of inflection point temperatures for LNP samples. The maximum slope of the melting curves shown over an8-degree temperature range in Figure S6 was calculated and the center temperature of that slope recorded.


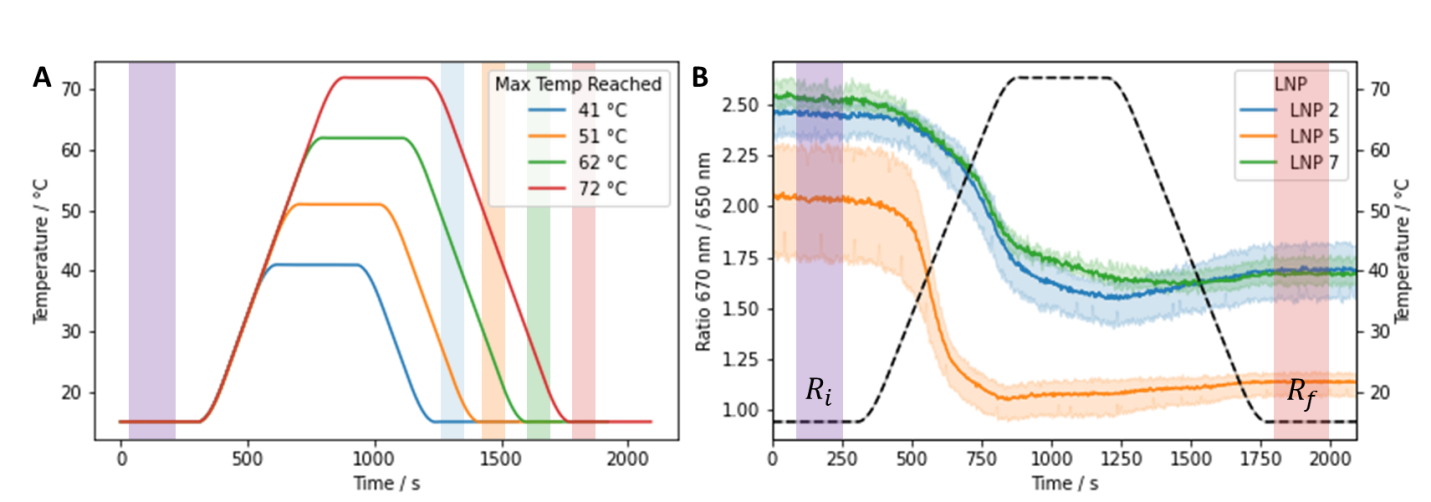


**Figure S9A**) Plot to show the temperature vs time of each stress testing experiment. **B)**Example traces for LNPs in PBS heated to 72 °C, with the regions for calculating Δ indicated.


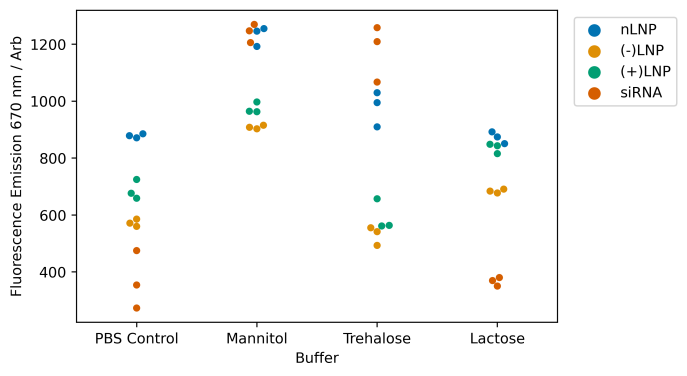

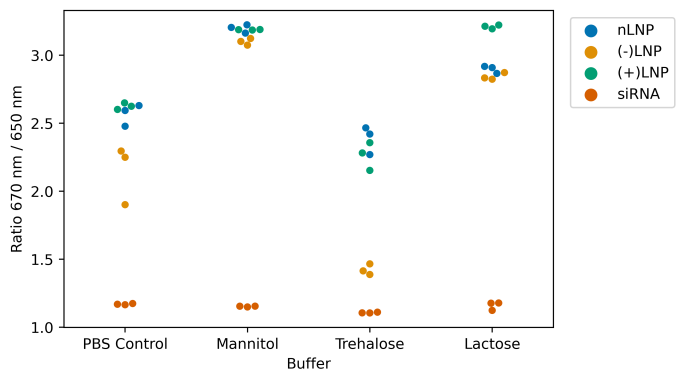


**Figure S10**Swarm plots of **A)** Initial Fluorescence and **B)** Initial Ratio of LNPs and Cy5-siRNA controls in buffers, as indicated. Data collected at 15 °C prior to heating the samples.

**
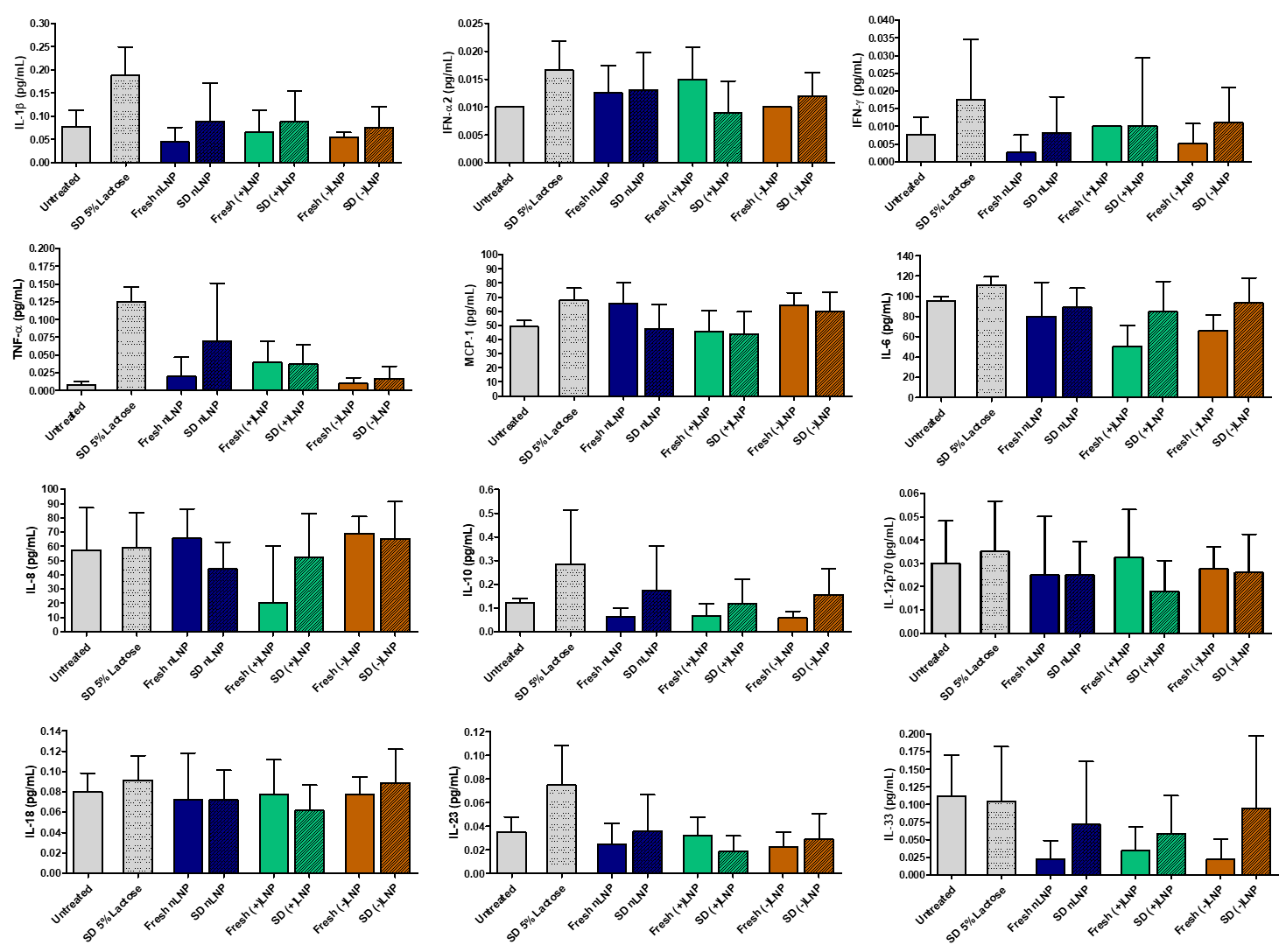
**

**Figure S11**Ex vivocytokinesecretion fromhPCLS transfected at 10 µg siRNA / mL with spray dried LNPs. Comparison of different cytokines after transfection with fresh and spray-dried (SD) LNPs. Mean ± standard deviation, n=3.
